# Plasma oxysterols are associated with serum lipids and dementia risk in older women

**DOI:** 10.1002/alz.13811

**Published:** 2024-04-04

**Authors:** Michelle M. Dunk, Stephen R. Rapp, Kathleen M. Hayden, Mark A. Espeland, Ramon Casanova, JoAnn E. Manson, Aladdin H. Shadyab, Robert Wild, Ira Driscoll

**Affiliations:** ^1^ Department of Psychology University of Wisconsin – Milwaukee Milwaukee Wisconsin USA; ^2^ Aging Research Center Department of Neurobiology, Care Sciences and Society Karolinska Institutet Stockholm Sweden; ^3^ Department of Social Sciences and Health Policy Division of Public Health Sciences Wake Forest University School of Medicine Winston‐Salem North Carolina USA; ^4^ Department of Psychiatry and Behavioral Medicine Wake Forest University School of Medicine Winston‐Salem North Carolina USA; ^5^ Department of Biostatistics and Data Science Wake Forest University School of Medicine Winston‐Salem North Carolina USA; ^6^ Sticht Center for Healthy Aging and Alzheimer's Prevention Wake Forest University School of Medicine Winston‐Salem North Carolina USA; ^7^ Department of Medicine Brigham and Women's Hospital, Harvard Medical School Boston Massachusetts USA; ^8^ Department of Epidemiology Harvard T.H. Chan School of Public Health Boston Massachusetts USA; ^9^ Herbert Wertheim School of Public Health and Human Longevity Science University of California San Diego La Jolla California USA; ^10^ Division of Geriatrics, Gerontology, and Palliative Care, Department of Medicine University of California San Diego La Jolla California USA; ^11^ Departments of Obstetrics and Gynecology, Biostatistics and Epidemiology Oklahoma University Health Sciences Center Oklahoma City Oklahoma USA; ^12^ Wisconsin Alzheimer's Disease Research Center University of Wisconsin‐Madison Madison Wisconsin USA

**Keywords:** 24‐hydroxycholesterol, 27‐hydroxycholesterol, Alzheimer's disease, Apolipoprotein E, cholesterol, dementia, mild cognitive impairment, oxysterols, Women's Health Initiative Memory Study

## Abstract

**INTRODUCTION:**

Apolipoprotein E4 (*APOE*4) carriers’ tendency toward hypercholesterolemia may contribute to Alzheimer's disease (AD) risk through oxysterols, which traverse the blood‐brain barrier.

**METHODS:**

Relationships between baseline plasma oxysterols, *APOE* status, serum lipids, and cognitive impairment risk were examined in 328 postmenopausal women from the Women's Health Initiative Memory Study. Women were followed for 25 years or until incident dementia or cognitive impairment.

**RESULTS:**

Levels of 24(S)‐hydroxycholesterol (24‐OHC), 27‐hydroxycholesterol (27‐OHC), and 24‐OHC/27‐OHC ratio did not differ by *APOE* status (*p*’s > 0.05). Higher 24‐OHC and 27‐OHC were associated with higher total, low density lipoprotein (LDL), non‐high density lipoprotein (HDL), remnant, LDL/HDL, and total/HDL cholesterol and triglycerides (*p*’s < 0.05). Higher 24‐OHC/27‐OHC was associated with greater dementia risk (hazard ratio = 1.51, 95% confidence interval:1.02‐2.22), which interaction analyses revealed as significant for *APOE*3 and *APOE*4+, but not *APOE*2+ carriers.

**DISCUSSION:**

Less favorable lipid profiles were associated with higher oxysterol levels. A higher ratio of 24‐OHC/27‐OHC may contribute to dementia risk in *APOE*3 and *APOE*4+ carriers.

## 1 BACKGROUND

The Apolipoprotein E ε4 (*APOE*4) allele is the main genetic risk factor for Alzheimer's disease (AD).^1^, ^2^ *APOE*4 carriers often have unfavorable lipid profiles, increasing their likelihood of hypercholesterolemia.^3^, ^4^ Midlife hypercholesterolemia is associated with heightened AD risk,^5^ suggesting that *APOE*’s involvement in lipid metabolism may contribute to AD risk. The underlying mechanisms are unclear, however, as blood cholesterol does not permeate the blood‐brain barrier.^6^ The literature suggests that oxysterols, which are oxidized products of cholesterol, may be an intermediary between hypercholesterolemia and AD due to their ability to cross the blood‐brain barrier.^6^, ^7^

The growing investigation into altered lipid metabolism as a central aspect of AD has revealed the involvement of a number of oxysterols, of which 24(S)‐hydroxycholesterol (24‐OHC) and 27‐hydroxycholesterol (27‐OHC) are among the most investigated.^6^, ^7^, ^8^, ^9^, ^10^ 24‐OHC is produced in the brain and serves as a primary route for excess brain cholesterol to exit into the periphery.^11^, ^12^ 24‐OHC appears to prevent AD pathology^13^, ^14^, ^15^, ^16^ but may be harmful at high concentrations.^17^ 27‐OHC primarily originates from peripheral blood cholesterol or consumption of cholesterol‐containing foods and travels into the brain.^18^, ^19^, ^20^, ^21^ In contrast to 24‐OHC, 27‐OHC promotes AD pathology^9^, ^10^, ^16^, ^22^, ^23^ and may even inhibit the protective effects of 24‐OHC.^21^, ^24^ Compared to controls, AD patients have lower 24‐OHC and higher 27‐OHC levels in the brain,^6^, ^25^, ^26^ and higher levels of both in cerebrospinal fluid.^27^, ^28^ Literature on plasma oxysterols in AD, however, is less consistent,^6^ potentially due to changing levels throughout the disease course.^29^

Given that AD pathology begins years before symptom onset,^30^ departure from the normal range of oxysterol levels may be most noticeable before AD diagnosis. Investigations of plasma 24‐OHC and 27‐OHC preceding diagnosis are therefore needed to clarify their involvement in early disease processes. Additionally, examinations of plasma oxysterols in relation to blood lipids prior to AD incidence are needed to corroborate speculations that oxysterols are a mechanistic link between hypercholesteremia and AD.^6^, ^7^, ^10^ We previously found that less favorable serum lipid profiles were associated with greater risk for dementia and cognitive impairment in the Women's Health Initiative (WHI) Memory Study (WHIMS^31^, ^32^).^33^ In the present study, we sought to determine the potential involvement of oxysterols in lipid‐dementia associations by investigating relationships of plasma oxysterols with *APOE*, serum lipids, and incident dementia and cognitive impairment in WHIMS.

## 2 METHODS

### 2.1 Participants

WHIMS, which evaluated the effects of hormone therapy (HT) on incident dementia and cognitive impairment in postmenopausal women,^31^, ^32^ is an ancillary study of the WHI clinical trial of HT.^34^ The current sample included WHIMS participants with available plasma oxysterol measurements from baseline blood draw (*N* = 328), previously assayed by another WHI ancillary study.^35^ A total of 281 participants had baseline serum lipids, and 264 were genotyped for *APOE*. Participants were cognitively unimpaired at baseline based on Modified Mini‐Mental State Examination (3MS) scores.^31^, ^32^ All participants provided written informed consent, and approval was obtained from Institutional Review Boards of participating sites.

### 2.2 Measures

Plasma oxysterols and serum lipids were measured from baseline fasting blood samples, frozen and stored at −70°C.^35^ Measurement details are provided in Appendix A and were published previously.^35^, ^36^, ^37^ Briefly, plasma oxysterol concentrations (24‐OHC and 27‐OHC; ng/mL) were measured at the University of Texas Southwestern Medical Center, Department of Molecular Genetics (Dallas, TX) using high‐performance liquid chromatography‐mass spectrometry (HPLC‐MS).^36^, ^37^ Assays were performed using a tertiary Shimadzu LC‐20XR HPLC system (Shimadzu Scientific Instruments; Columbia, MD) coupled to an AB Sciex API‐5000 triple quadrupole MS equipped with Turbo V ESI source (Foster City, CA).^36^ The average interassay coefficients of variability (CVs) of 24‐OHC and 27‐OHC were 9.5% (maximum 30.7%) and 9.5% (maximum 36.6%), respectively.

Serum lipids (total, low density lipoprotein [LDL], and high density lipoprotein [HDL] cholesterol) [mg/dL] and triglycerides [mg/dL]) were measured at the University of Minnesota Medical Center Advanced Research and Diagnostics Lab. The Roche Modular P Chemistry analyzer (Roche Diagnostics Corporation, Indianapolis, IN 46250) was used to determine total cholesterol, HDL cholesterol, and triglyceride levels. The Friedewald formula was used to calculate LDL cholesterol levels.^38^ From these, we calculated non‐HDL cholesterol (total cholesterol minus HDL cholesterol) and remnant cholesterol (total cholesterol minus LDL and HDL cholesterol) levels, as well as total/HDL and LDL/HDL ratios. Interassay CVs were: total cholesterol, 3.8% at 205.5 mg/dL and 5.1% at 252.0 mg/dL; HDL cholesterol, 3.4% at 29.2 mg/dL and 2.3% at 57.6 mg/dL; triglycerides, 2.0% at 85 mg/dL and 1.8% at 183 mg/dL.

*APOE* genotype was ascertained from blood samples using single nucleotide polymorphisms (SNPs) rs429358 and rs7412.^39^

Possible confounders collected at WHI enrollment included age, education, smoking status, weekly alcohol consumption, body mass index (BMI) measurement, WHI HT assignment, cholesterol‐lowering medication, hypertension, cardiovascular disease, type 2 diabetes, stroke, and dietary information (cholesterol, fat, protein, carbohydrate, and calorie intake).

Global cognitive function was assessed annually from 1995 to 2007 using the 3MS.^31^, ^32^ Between 2008 and 2021, annual cognitive assessment continued through the WHIMS Epidemiology of Cognitive Health Outcomes (WHIMS‐ECHO) study using a validated, telephone‐administered cognitive battery.^40^ Those scoring below age‐ and education‐adjusted cutoff points were evaluated further via the Consortium to Establish a Registry for Alzheimer's Disease (CERAD) neuropsychological battery,^41^ clinical evaluation, and proxy interviews.^31^, ^32^ A centralized panel of experts reviewed all information for adjudication of no impairment, probable dementia, or mild cognitive impairment (MCI; a prodrome of AD^42^) according to standardized diagnostic criteria (Diagnostic and Statistical Manual of Mental Disorders, Fourth Edition; MCI criteria defined by Petersen et al.).^31^, ^32^, ^42^

### 2.3 Statistical analysis

We classified participants as *APOE*2+ (ε2/ε2, ε2/ε3), *APOE*3 (ε3/ε3), or *APOE*4+ (ε3/ε4, ε4/ε4) carriers for analyses. *APOE* ε2/ε4 carriers (*N* = 5) were excluded due to small sample size and opposing effects of ε2 and ε4 alleles on lipid metabolism and dementia risk.^1^, ^2^, ^3^, ^4^

**RESEARCH IN CONTEXT**

1. **Systematic review**: Literature on oxysterols concerning Alzheimer's disease (AD), blood lipids, and apolipoprotein E (*APOE)* was reviewed. Midlife hypercholesterolemia is associated with greater AD risk, and *APOE*4 carriers are at increased risk for both. Literature suggests oxysterols as an intermediary between hypercholesterolemia and AD. While 24‐OHC and 27‐OHC levels in the brain and cerebrospinal fluid are altered in AD, relationships between oxysterols and blood lipids, *APOE*, and AD are still not well understood.
2. **Interpretation**: A higher plasma 24‐OHC/27‐OHC ratio is associated with greater dementia risk in *APOE*4+ and *APOE*3 carriers. Less favorable lipid levels are associated with higher 24‐OHC and 27‐OHC, suggesting that plasma oxysterols may be a modifiable risk factor for dementia.
3. **Future directions**: Future research using larger samples is required to determine if midlife hypercholesterolemia alters subsequent oxysterol levels, and whether oxysterol modification through lipid management can reduce AD risk.

Multiple linear regression was performed to investigate associations of plasma oxysterols with *APOE* (*APOE*3 as reference) and serum lipids. Regression models were initially adjusted for age, BMI, WHI HT assignment, and cholesterol‐lowering medication, followed by further adjustment for education, smoking, alcohol intake, hypertension, cardiovascular disease, and diabetes. Cox proportional hazards regression was performed to examine incident dementia and cognitive impairment (dementia+MCI) in relation to plasma oxysterols. We also examined interactions between oxysterols and *APOE* status in relation to cognitive outcomes. Participants were followed from baseline visit to either the time of MCI or dementia diagnosis (dementia diagnosis for those who progressed from MCI to dementia), loss to follow‐up, or end of follow‐up, whichever came first. Cox regression models were adjusted for age, BMI, HT assignment, and cholesterol‐lowering medication. Additional adjustments were performed for education, smoking, alcohol consumption, hypertension, cardiovascular disease, diabetes, stroke, total serum cholesterol, serum triglycerides, total dietary cholesterol intake, total calorie intake, total fat intake, total protein intake, and total carbohydrate intake. Power calculations were performed for all analyses due to the relatively small sample size (details and results [Tables A1 and A2] can be found in Appendix A).

An increased risk for dementia and cognitive impairment was previously reported in relation to estrogen and estrogen plus progestin therapy in WHIMS.^31^, ^32^ We therefore performed supplementary analyses of all associations stratified by HT assignment to assess for possible effect modification by hormone therapy.

Analyses were performed using SAS® Studio on the SAS OnDemand for Academics platform, version 3.8 (©2012‐2018, SAS Institute Inc, Cary, NC) with significance reported at *p *< 0.05. Histograms and scatterplots of oxysterols were created in R Studio version 2023.06.1 + 524, © 2009‐2023, Posit Software, PBC.

## 3 RESULTS

### 3.1 Sample characteristics

After a mean follow‐up of 9.39 (SD = 6.07) years between 1995 and 2021 (median = 7.00 [IQR = 5.00] years until censoring for those lost to follow‐up), 35 (11%) participants were classified with probable dementia (*APOE*2+, *n* = 4; *APOE*3, *n* = 18; *APOE*4+, *n* = 11; missing *APOE* status, *n* = 2) and 61 (19%) with cognitive impairment (*APOE*2+, *n* = 6; *APOE*3, *n* = 29; *APOE*4+, *n* = 19; missing *APOE* status, *n* = 7), while 267 (81%) remained cognitively unimpaired. The mean age at enrollment was 71.52 (SD = 4) years. There were 39 (15%) *APOE*2+, 166 (63%) *APOE*3, and 59 (22%) *APOE*4+ carriers. Baseline characteristics are reported in Table 1. Histograms showing the distribution of plasma oxysterols are displayed in Figure A1 (Appendix A).

**TABLE 1:** Baseline characteristics by diagnostic classification

	Cognitive outcome			
Characteristic	Unimpaired (*N* = 267)	MCI (*N* = 26)	Dementia (*N* = 35)	*P*‐Value
Age (years), M (SD)	71.5 (4.0)	71.5 (4.5)	71.8 (3.6)	0.89
Education, N (%) (missing = 2)				0.26
<High school	18 (6.7)	1 (3.8)	2 (5.7)	
High school	61 (22.8)	4 (15.4)	8 (22.9)	
College	136 (50.9)	13 (50.0)	15 (42.9)	
Postgraduate	50 (18.7)	8 (30.8)	10 (28.6)	
Smoking, N (%) (missing = 2)				0.73
Never	137 (51.3)	14 (53.8)	19 (54.3)	
Former	110 (41.2)	11 (42.3)	14 (40.0)	
Current	18 (6.7)	1 (3.8)	1 (2.9)	
Alcohol, servings per week, M (SD)	2.6 (5.7)	1.8 (3.9)	1.3 (2.5)	0.32
Serum lipids, mg/dL, M (SD) (missing = 47)				
LDL cholesterol	149.4 (34.8)	164.7 (33.9)	148.5 (35.1)	0.13
HDL cholesterol	52.9 (12.4)	53.6 (9.6)	52.8 (11.5)	0.96
Total cholesterol	229.8 (38.1)	245.1 (35.5)	228.9 (40.1)	0.18
Non‐HDL cholesterol	177.0 (38.9)	191.5 (35.6)	176.1 (38.7)	0.22
Remnant cholesterol	27.5 (13.5)	26.9 (14.2)	27.6 (13.7)	0.98
LDL/HDL ratio	3.0 (1.0)	3.2 (0.9)	2.9 (0.8)	0.59
Total/HDL ratio	4.6 (1.3)	4.7 (1.2)	4.5 (1.1)	0.76
Triglycerides	138.1 (67.8)	134.0 (70.6)	138.0 (68.4)	0.96
Plasma oxysterols, ng/mL, M (SD)				
24‐OHC	65.7 (20.0)	68.3 (18.8)	67.9 (17.9)	0.70
27‐OHC	152.6 (45.1)	156.9 (46.7)	147.2 (30.2)	0.68
24‐OHC/27‐OHC ratio	0.4 (0.1)	0.5 (0.1)	0.5 (0.1)	0.32
Total energy intake, kcal/day, M (SD)	1526.8 (634.4)	1389.0 (637.2)	1621.4 (572.7)	0.36
Dietary cholesterol, g/day, M (SD)	209.7 (125.9)	174.9 (99.7)	195.4 (105.8)	0.34
Dietary carbohydrate, g/day, M (SD)	184.0 (77.5)	179.1 (86.3)	211.2 (74.5)	0.14
Dietary protein, g/day, M (SD)	63.8 (29.2)	60.5 (31.6)	63.1 (22.8)	0.85
Dietary total fat, g/day, M (SD)	58.2 (30.6)	48.2 (24.9)	60.5 (32.6)	0.23
Body mass index, kg/m^2^, M (SD) (missing = 1)	27.5 (5.1)	27.5 (5.1)	27.1 (3.9)	0.92
*APOE* status (missing = 64)				0.07
*APOE*2+	33 (12.4)	2 (7.7)	4 (11.4)	
*APOE*3	137 (51.3)	11 (42.3)	18 (51.4)	
*APOE*4+	40 (15.0)	8 (30.8)	11 (31.4)	
Cholesterol‐lowering medication, N (%)	36 (13.5)	4 (15.4)	2 (5.7)	0.40
Hypertension, N (%)	107 (40.1)	10 (38.5)	10 (28.6)	0.39
Cardiovascular disease, N (%)	55 (20.6)	7 (26.9)	5 (14.3)	0.49
Stroke, N(%)	2 (0.7)	0 (0)	0 (0)	0.78
Diabetes, N(%)	20 (7.5)	1 (3.8)	1 (2.9)	0.49
Fracture, N(%)	170 (63.7)	12 (46.2)	20 (57.1)	0.18

*Note*: SI conversion factor: To convert cholesterol levels to mmol/L, multiply values by 0.0259. To convert triglyceride levels to mmol/L, multiply values by 0.0113. To convert oxysterols to nmol/L, multiply values by 2.48.

Abbreviations: 24‐OHC, 24(S)‐hydroxycholesterol; 27‐OHC, 27‐hydroxycholesterol; HDL, high‐density lipoprotein; LDL, low‐density lipoprotein; M, mean; MCI, mild cognitive impairment; SD, standard deviation.

### 3.2 Relationships between oxysterols, *APOE*, and blood lipids

Plasma oxysterols did not significantly differ by *APOE* status (*p*’s > 0.11), albeit higher in *APOE*4+ and *APOE*3 compared to *APOE*2+ carriers (Table A3 and Figure A2, Appendix A).

Higher levels of LDL (β = 0.24 [95% confidence interval [CI]:0.19‐0.30]), total (β = 0.23 [CI:0.18‐0.28]), non‐HDL (β = 0.24 [CI:0.19‐0.28]), remnant (β = 0.34 [CI:0.18‐0.49]), LDL/HDL (β = 6.91 [CI:4.78‐9.04]), and total/HDL cholesterol (β = 5.26 [CI:3.55‐6.96]), and triglycerides (β = 0.07 [CI:0.04‐0.10]) were associated with higher 24‐OHC (*p*’s < 0.001; Table 2; Figures A3–A5 in Appendix A). Higher levels of LDL (β = 0.68 [CI:0.57‐0.78]), total (β = 0.63 [CI:0.53‐0.72]), non‐HDL (β = 0.63 [CI:0.53‐0.72]), remnant (β = 0.72 [CI:0.37‐1.06]), LDL/HDL (β = 18.05 [CI:13.60‐22.51]), and total/HDL cholesterol (β = 13.31 [CI:9.70‐16.91]), and triglycerides (β = 0.14 [CI:0.07‐0.21]) were associated with higher 27‐OHC (*p*’s < 0.001). Serum lipids were not associated with 24‐OHC/27‐OHC ratio (*p*’s > 0.26).

**TABLE 2:** Associations between oxysterols and lipids

	Limited adjustment**^a^**		Full adjustment**^b^**	
Lipid	β (95% CI)	*P*‐Value	β (95% CI)	*P*‐Value
24‐OHC				
LDL cholesterol	0.25 (0.20, 0.31)	<0.0001	0.24 (0.19, 0.30)	<0.0001
HDL cholesterol	−0.11 (−0.30, 0.07)	0.23	−0.09 (−0.28, 0.10)	0.35
Total cholesterol	0.24 (0.19, 0.29)	<0.0001	0.23 (0.18, 0.28)	<0.0001
Non‐HDL cholesterol	0.25 (0.20, 0.30)	<0.0001	0.24 (0.19, 0.28)	<0.0001
Remnant cholesterol	0.36 (0.20, 0.51)	<0.0001	0.34 (0.18, 0.49)	<0.0001
LDL/HDL ratio	7.44 (5.34, 9.55)	<0.0001	6.91 (4.78, 9.04)	<0.0001
Total/HDL ratio	5.61 (3.93, 7.29)	<0.0001	5.26 (3.55, 6.96)	<0.0001
Triglycerides	0.07 (0.04, 0.10)	<0.0001	0.07 (0.04, 0.10)	<0.0001
27‐OHC				
LDL cholesterol	0.67 (0.57, 0.77)	<0.0001	0.68 (0.57, 0.78)	<0.0001
HDL cholesterol	−0.10 (−0.49, 0.29)	0.61	−0.06 (−0.47, 0.35)	0.77
Total cholesterol	0.62 (0.53, 0.71)	<0.0001	0.63 (0.53, 0.72)	<0.0001
Non‐HDL cholesterol	0.63 (0.54, 0.72)	<0.0001	0.63 (0.53, 0.72)	<0.0001
Remnant cholesterol	0.68 (0.35, 1.01)	<0.0001	0.72 (0.37, 1.06)	<0.0001
LDL/HDL ratio	18.61 (14.34, 22.87)	<0.0001	18.05 (13.60, 22.51)	<0.0001
Total/HDL ratio	13.50 (10.05, 16.96)	<0.0001	13.31 (9.70, 16.91)	<0.0001
Triglycerides	0.14 (0.07, 0.20)	<0.0001	0.14 (0.07, 0.21)	<0.0001
24‐OHC/27‐OHC				
LDL cholesterol	−0.0002 (−0.001, 0.0002)	0.34	−0.0002 (−0.001, 0.0002)	0.27
HDL cholesterol	0.0001 (−0.001, 0.001)	0.88	0.0001 (−0.001, 0.001)	0.86
Total cholesterol	0.0001 (−0.0004, 0.0002)	0.53	−0.0002 (−0.001, 0.0002)	0.39
Non‐HDL cholesterol	−0.0001 (−0.001, 0.0002)	0.50	−0.0002 (−0.001, 0.0002)	0.37
Remnant cholesterol	0.0003 (−0.001, 0.001)	0.57	0.0001 (−0.001, 0.001)	0.81
LDL/HDL ratio	−0.01 (−0.02, 0.01)	0.35	−0.01 (−0.02, 0.01)	0.32
Total/HDL ratio	−0.004 (−0.01, 0.01)	0.49	−0.004 (−0.02, 0.01)	0.45
Triglycerides	0.0001 (−0.0001, 0.0003)	0.57	0.00 (−0.0002, 0.0002)	0.81

Abbreviations: 24‐OHC, 24(S)‐hydroxycholesterol; 27‐OHC, hydroxycholesterol; 95% CI, 95% confidence interval; BMI, body mass index; HDL, high‐density lipoprotein; HT, hormone therapy; LDL, low‐density lipoprotein; LSM, least squares means; SE, standard error.

^a^ Age, BMI, cholesterol‐lowering medication, and HT trial assignment.

^b^ Age, BMI, cholesterol‐lowering medication, HT trial assignment, education, hypertension, cardiovascular disease, diabetes, smoking, and weekly alcohol consumption.

### 3.3 Relationships between oxysterols and dementia risk

Higher 24‐OHC/27‐OHC ratio was associated with greater dementia risk (fully adjusted hazard ratio [HR] = 1.51 [CI:1.02‐2.22], *p *= 0.04; Table 3), but neither 24‐OHC nor 27‐OHC alone was associated with dementia risk (*p*’s > 0.14). Relationships between oxysterols and cognitive impairment did not reach significance (*p*’s > 0.25).

**TABLE 3:** Associations between oxysterols and dementia risk

	Limited adjustment**^a^**		Full adjustment**^b^**	
Cognitive Outcome	Hazard ratio (95% CI)	*P*‐Value	Hazard ratio (95% CI)	*P*‐Value
24‐OHC				
Dementia	1.01 (0.99, 1.03)	0.25	1.02 (0.99, 1.05)	0.15
Cognitive impairment	1.01 (0.99, 1.02)	0.26	1.01 (0.99, 1.03)	0.38
27‐OHC				
Dementia	1.00 (0.99, 1.01)	0.73	1.00 (0.98, 1.02)	0.90
Cognitive impairment	1.00 (0.99, 1.01)	0.96	1.00 (0.99, 1.01)	0.85
24‐OHC/27‐OHC				
Dementia	1.35 (1.02, 1.79)	0.04	1.51 (1.02, 2.22)	0.04
Cognitive impairment	1.23 (0.98, 1.55)	0.07	1.23 (0.91, 1.66)	0.18

*Note*: Hazard ratios of oxysterol levels were calculated per 1 ng/mL increase in 24‐OHC and 27‐OHC levels, and per 0.1 unit increase in 24‐OHC/27‐OHC ratio.

Abbreviations: 24‐OHC, 24(S)‐hydroxycholesterol; 27‐OHC, 27‐hydroxycholesterol; 95% CI, 95% confidence interval; BMI, body mass index; HT, hormone therapy.

^a^ Age, BMI, cholesterol‐lowering medication, and HT trial assignment.

^b^ Age, BMI, cholesterol‐lowering medication, HT trial assignment, education, cardiovascular disease, hypertension, stroke, diabetes, weekly alcohol consumption, smoking status, dietary cholesterol intake, total protein intake, total carbohydrate intake, total fat intake, total caloric intake, total blood cholesterol, and triglycerides.

Further analysis assessing interactions between oxysterols and *APOE* revealed lower risk of dementia (fully adjusted HR = 0.02 [CI:0.00‐0.71], *p *= 0.02) and cognitive impairment (fully adjusted HR = 0.04 [CI:0.00‐0.78], *p *= 0.03) in relation to higher 24‐OHC/27‐OHC in *APOE*2+ compared to *APOE*3 carriers (Table 4; Figure A6 in Appendix A).

**TABLE 4:** Interactions between oxysterols and *APOE* in relation to dementia risk

		Limited adjustment**^a^**	Full adjustment**^b^**
Cognitive Outcome	*APOE ×* oxysterol interaction	Hazard ratio (95% CI)	Hazard ratio (95% CI)
Dementia	*APOE ×* 24‐OHC		
	*APOE*2+	0.97 (0.91, 1.03)	0.96 (0.87, 1.05)
	*APOE*4+	1.01 (0.99, 1.04)	1.02 (0.99, 1.06)
	*APOE ×* 27‐OHC		
	*APOE*2+	0.99 (0.97, 1.02)	1.00 (0.96, 1.03)
	*APOE*4+	1.00 (0.98, 1.02)	1.00 (0.98, 1.02)
	*APOE ×* 24‐OHC/27‐OHC		
	*APOE*2+	0.22 (0.02, 2.57)	0.02^*^ (0.00, 0.71)
	*APOE*4+	1.39 (0.95, 2.04)	1.83 (1.07, 3.13)
Cognitive impairment	*APOE ×* 24‐OHC		
	*APOE*2+	0.97 (0.92‐1.04)	0.96 (0.90, 1.03)
	*APOE*4+	1.02 (1.00, 1.04)	1.01 (0.98, 1.04)
	*APOE ×* 27‐OHC		
	*APOE*2+	0.99 (0.97, 1.01)	0.99 (0.97, 1.02)
	*APOE*4+	1.01 (0.99, 1.02)	1.00 (0.98, 1.01)
	*APOE ×* 24‐OHC/27‐OHC		
	*APOE*2+	0.23 (0.03, 1.73)	0.04^*^ (0.00, 0.78)
	*APOE*4+	1.32 (0.96, 1.80)	1.42 (0.96, 2.10)

*Note*: Hazard ratios of oxysterol levels were calculated per 1 ng/mL increase in 24‐OHC and 27‐OHC levels, and per 0.1 unit increase in 24‐OHC/27‐OHC ratio.

Abbreviations: 24‐OHC, 24(S)‐hydroxycholesterol; 27‐OHC, 27‐hydroxycholesterol; 95% CI, 95% confidence interval; BMI, body mass index; HT, hormone therapy; x, interaction.

^a^ Age, BMI, cholesterol‐lowering medication, and HT trial assignment.

^b^ Age, BMI, cholesterol‐lowering medication, HT trial assignment, education, cardiovascular disease, hypertension, stroke, diabetes, weekly alcohol consumption, smoking status, dietary cholesterol intake, total protein intake, total carbohydrate intake, total fat intake, total caloric intake, total blood cholesterol, and triglycerides.

* *p *< 0.05.

### 3.4 Supplementary analysis

Results from supplementary analyses are detailed in Appendix A (Tables A4‐A6). Briefly, associations of oxysterols with *APOE* and serum lipids were largely similar to those in the entire sample, regardless of HT. There were some differences in associations between oxysterols and cognitive impairment based on HT groups, but many models failed to converge and these findings should be interpreted with caution given the limited statistical power due to small sample sizes.

## 4 DISCUSSION

In this prospective cohort of postmenopausal women, we report: (1) higher plasma 24‐OHC and 27‐OHC in relation to higher lipid levels, and (2) greater dementia and cognitive impairment risk in relation to a higher ratio of 24‐OHC/27‐OHC in *APOE*4+ and *APOE*3 carriers.

The present study is not alone in reporting a lack of variation in oxysterols by *APOE* status. No *APOE*‐related differences in serum 24‐OHC were reported in a sample of 80 AD patients,^43^ although another sample of 53 AD patients found lower plasma 24‐OHC in *APOE*4 compared to noncarriers.^29^ Literature on oxysterol‐*APOE* relationships remains limited, especially in cognitively unimpaired older adults. Our sample, while one of the larger examined to date, is still relatively small for genetic analyses. Investigations in much larger samples will be necessary to understand *APOE*‐related variation in oxysterols with some certainty.

The associations between less favorable lipids and higher 24‐OHC and 27‐OHC are consistent with some of the literature,^6^, ^9^, ^19^, ^20^, ^21^, ^44^ although a recent meta‐analysis of case‐control studies reported no differences in 24‐OHC between MCI (*n* = 260) or AD (*n* = 509) patients and controls (*n* = 438 and *n* = 428, respectively).^8^ Higher dementia risk observed in those with a higher 24‐OHC/27‐OHC ratio is in agreement with the marginally significant findings of increased cognitive impairment risk reported by Hughes et al., but, in contrast, we did not observe a relationship between 24‐OHC levels and dementia risk in our exclusively female sample.^45^ Nonetheless, our study is the first to report significantly greater dementia risk in relation to higher 24‐OHC/27‐OHC, and specifically in *APOE*3 and *APOE*4+ carriers. While this finding could reflect a protective effect of a higher 24‐OHC/27‐OHC ratio against dementia among *APOE*2+ carriers, the consistent reports of the deleterious effects of altered oxysterol levels^6^, ^7^, ^8^, ^9^, ^10^, ^16^, ^17^, ^21^, ^22^, ^23^, ^24^ suggest a greater likelihood that *APOE* ε2 allele may instead be protective against pathological effects of abnormal oxysterol levels. Nevertheless, this interaction between oxysterols and *APOE* in relation to cognitive impairment should be interpreted with caution given the small number of cases among *APOE*2+ carriers. We did not observe greater dementia risk for *APOE*4+ compared to *APOE*3 carriers in relation to 24‐OHC/27‐OHC ratio, which also raises questions and further necessitates examination in larger samples to clarify *APOE*‐oxysterol relationships.

Overall, our results align with discussions in the literature regarding alterations in oxysterol levels along the AD trajectory. Hypercholesterolemia likely increases 27‐OHC levels,^6^, ^7^, ^12^, ^19^, ^20^, ^21^, ^44^ which is consistent with the positive association between lipids and 27‐OHC in this cohort. High amounts of 27‐OHC may then enter the brain, promote AD pathology, and drive out 24‐OHC—resulting in subsequent elevation of 24‐OHC/27‐OHC levels in the peripheral circulation.^6^, ^7^, ^9^, ^10^, ^16^, ^18^, ^20^, ^21^, ^24^ WHIMS participants were ≥65 years at the time of oxysterol collection, by which time those who were hypercholesterolemic in midlife would presumably have both a higher ratio of plasma 24‐OHC/27‐OHC and greater dementia risk—a hypothesis that seems consistent with our findings. If the observed increase in dementia risk in relation to higher plasma 24‐OHC/27‐OHC in older age is in fact a reflection of this proposed sequence of events, our findings may be in agreement with prior reports of 27‐OHC as an instigator of AD pathology and an inhibitor of 24‐OHC's ability to safeguard against AD pathology.^6^, ^7^, ^9^, ^10^, ^16^, ^21^, ^22^, ^23^, ^24^ To confirm whether abnormal oxysterol levels serve as a mechanistic link between hypercholesterolemia and AD will require investigations in larger samples with available midlife lipid levels, subsequent oxysterol levels, and AD incidence.

If our findings can be confirmed in larger samples, this would suggest the possibility of dementia risk modification via oxysterol management. Dietary patterns that are high in whole, minimally processed plant foods have been shown to promote healthy blood lipids,^46^ which may prevent elevated 27‐OHC levels.^6^, ^7^, ^12^, ^19^, ^20^, ^21^, ^43^, ^47^ Consumption of cholesterol‐containing foods, especially when the cholesterol has been oxidized from exposure to oxygen, sunlight, or high temperatures, is also associated with higher 27‐OHC.^19^, ^20^, ^21^ Vascular risk management including dietary and pharmacological interventions appears to reduce 27‐OHC and 24‐OHC blood levels,^43^, ^48^ but further research is required to determine whether such interventions could be effective in reducing dementia risk.

Study limitations include an exclusively female sample which is relatively small due to limited oxysterol collection. Those selected for oxysterol analysis by Chang et al. included 872 WHI HT participants—442 with incident hip or vertebral fracture during follow‐up, and 430 randomly selected participants with sufficient volumes of prerandomization plasma sample.^35^ Although this previous study's selection criteria limited our sample, fracture risk in the current study did not differ by cognitive outcomes (Table 1) and therefore should not have influenced our findings. WHIMS assessed for dementia due to all causes.^31^, ^32^ While AD was the most common dementia classification,^31^, ^32^ the potential inclusion of several vascular dementia cases (the second most common cause of dementia after AD^30^, ^49^) could have led to underestimation of the associations between oxysterols and cognitive outcomes. Additionally, this sample did not have midlife lipid levels, preventing us from analyzing prior hypercholesterolemia in relation to subsequent oxysterol levels. Nonetheless, WHIMS is a well‐screened, prospectively followed sample. To our knowledge, this is the first study to comprehensively examine plasma oxysterols in older adults in relation to *APOE*, a large lipid panel, and adjudicated incident dementia and cognitive impairment.

Overall, higher 24‐OHC and 27‐OHC are associated with less favorable lipid profiles, and *APOE*3 and *APOE*4+ carriers with a higher 24‐OHC/27‐OHC ratio may be at greater risk for dementia and cognitive impairment. These findings offer support for the involvement of 24‐OHC and 27‐OHC in dementia, motivating further research into the clinical significance of oxysterols as modifiable factors contributing to AD risk. Whether AD risk reduction is possible through the maintenance of optimal oxysterol levels warrants further investigation.

## CONFLICT OF INTEREST STATEMENT

The authors report no conflicts of interest. Author disclosures are available in the supporting information.

## Supporting information

Supporting Information

Supporting Information

Supporting Information

## References

[1] Serrano‐Pozo A , Das S , Hyman BT . APOE and Alzheimer's disease: advances in genetics, pathophysiology, and therapeutic approaches. Lancet Neurol. 2021;20(1):68‐80. doi:10.1016/S1474-4422(20)30412-9 33340485 PMC8096522

[2] Chartier‐Harlin M‐C , Parfitt M , Legrain S , et al. Apolipoprotein E, ε4 allele as a major risk factor for sporadic early and late‐onset forms of Alzheimer's disease: analysis of the 19q13.2 chromosomal region. Hum Mol Genet. 1994;3(4):569‐574. 8069300 10.1093/hmg/3.4.569

[3] Boerwinkle E , Sing CF . The use of measured genotype information in the analysis of quantitative phenotypes in man. III. Simultaneous estimation of the frequencies and effects of the apolipoprotein E polymorphism and residual polygenetic effects on cholesterol, betalipoprotein and triglyceride levels. Ann Hum Genet. 1987;51(3):211‐226. doi:10.1111/j.1469-1809.1987.tb00874.x 3688836

[4] Bennet AM , Di Angelantonio E , Ye Z , et al. Association of apolipoprotein E genotypes with lipid levels and coronary risk. JAMA. 2007;298(11):1300‐1311. doi:10.1001/jama.298.11.1300 17878422

[5] Anstey KJ , Ashby‐Mitchell K , Peters R . Updating the evidence on the association between serum cholesterol and risk of late‐life dementia: review and meta‐analysis. J Alzheimers Dis. 2017;56:215‐228. doi:10.3233/JAD-160826 27911314 PMC5240556

[6] Gamba P , Testa G , Gargiulo S , Staurenghi E , Poli G , Leonarduzzi G . Oxidized cholesterol as the driving force behind the development of Alzheimer's disease. Front Aging Neurosci. 2015;7:1‐21. doi:10.3389/fnagi.2015.00119 26150787 PMC4473000

[7] Björkhem I . Crossing the barrier: oxysterols as cholesterol transporters and metabolic modulators in the brain. J Internal Med. 2006;260:493‐508. doi:10.1111/j.1365-2796.2006.01725.x 17116000

[8] Ademowo OS , Dias IHK . Circulating oxysterols in Alzheimer's disease: a systematic review and meta‐analysis. Redox Exp Med. 2022;2022(1):R116‐R126. doi:10.1530/REM-22-0009

[9] Leoni V , Caccia C . Potential diagnostic applications of side chain oxysterols analysis in plasma and cerebrospinal fluid. Biochem Pharmacol. 2013;86:26‐36. doi:10.1016/j.bcp.2013.03.015 23541982

[10] Marwarha G , Ghribi O . Does the oxysterol 27‐hydroxycholesterol underlie Alzheimer's disease‐Parkinson's disease overlap? Exp Gerontol. 2015;68:13‐18. doi:10.1016/j.exger.2014.09.013 25261765 PMC4377123

[11] Lütjohann D , Breuer O , Ahlborg G , et al. Cholesterol homeostasis in human brain: evidence for an age‐dependent flux of 24S‐hydroxycholesterol from the brain into the circulation. Proc Natl Acad Sci USA. 1996;93:9799‐9804. 8790411 10.1073/pnas.93.18.9799 PMC38509

[12] Björkhem I , Lütjohann D , Diczfalusy U , et al. Cholesterol homeostasis in human brain: turnover of 24S‐hydroxycholesterol and evidence for a cerebral origin of most of this oxysterol in the circulation. J Lipid Res. 1998;39(8):1594‐1600. 9717719

[13] Bryleva EY , Rogers MA , Chang CCY , et al. ACAT1 gene ablation increases 24(S)‐hydroxycholesterol content in the brain and ameliorates amyloid pathology in mice with AD. PNAS. 2010;107(7):3081‐3086. doi:10.1073/pnas.0913828107 20133765 PMC2840286

[14] Hudry E , Van Dam D , Kulik W , et al. Adeno‐associated virus gene therapy with cholesterol 24‐hydroxylase reduce the amyloid pathology before or after the onset of amyloid plaques in mouse models of Alzheimer's disease. Mol Ther. 2010;18(1):44‐53. doi:10.1038/mt.2009.175 19654569 PMC2839219

[15] Testa G , Staurenghi E , Giannelli S , et al. A silver lining for 24‐hydroxycholesterol in Alzheimer's disease: the involvement of the neuroprotective enzyme sirtuin 1. Redox Biol. 2018;17:423‐431. doi:10.1016/j.redox.2018.05.009 29883958 PMC6007083

[16] Prasanthi JRP , Huls A , Thomasson S , Thompson A , Schommer E , Ghribi O . Differential effects of 24‐hydroxycholesterol and 27‐hydroxycholesterol on β‐amyloid precursor protein levels and processing in human neuroblastoma SH‐SY5Y cells. Mol Neurodegener. 2009;4(1):1‐8. doi:10.1186/1750-1326-4-1 19126211 PMC2654562

[17] Gamba P , Giannelli S , Staurenghi E , et al. The controversial role of 24‐S‐hydroxycholesterol in Alzheimer's disease. Antioxidants. 2021;10:740. doi:10.3390/antiox10050740 34067119 PMC8151638

[18] Heverin M , Meaney S , Lütjohann D , Diczfalusy U , Wahren J , Bjorkhem I . Crossing the barrier: net flux of 27‐hydroxycholesterol into the human brain. J Lipid Res. 2005;46:1047‐1052. doi:10.1194/jlr.M500024-JLR200 15741649

[19] Otaegui‐Arrazola A , Menendez‐Carreno M , Ansorena D , Astiasaran I . Oxysterols: a world to explore. Food Chem Toxicol . 2010;48:3289‐3303. doi:10.1016/j.fct.2010.09.023 20870006

[20] Brooks SW , Dykes AC , Schreurs BG . A high‐cholesterol diet increases 27‐hydroxycholesterol and modifies estrogen receptor expression and neurodegeneration in rabbit hippocampus. J Alzheimers Dis. 2017;56(1):185‐196. doi:10.3233/JAD-160725 27911307 PMC5382498

[21] Liu Q , An Y , Ma W , et al. High‐cholesterol diet results in elevated amyloid‐β and oxysterols in rats. Mol Med Rep. 2018;17:1235‐1240. doi:10.3892/mmr.2017.8003 29115521

[22] Glöckner F , Meske V , Lütjohann D , Ohm TG . Dietary cholesterol and its effect on tau protein: a study in apolipoprotein E‐deficient and P301L human tau mice. J Neuropathol Exp Neurol. 2011;70(4):292‐301. doi:10.1097/NEN.0b013e318212f185 21412171

[23] Wang Z‐H , Xia Y , Liu P , et al. ApoE4 activates C/EBPβ/δ‐secretase with 27‐hydroxycholesterol, driving the pathogenesis of Alzheimer's disease. Prog Neurobiol. 2021;202:102032. doi:10.1016/j.pneurobio.2021.102032 33716161 PMC8627566

[24] Zhang X , Xi Y , Yu H , et al. 27‐hydroxychoelsterol promotes Aβ accumulation via altering Aβ metabolism in mild cognitive impairment patients and APP/PS1 mice. Brain Pathol. 2019;29(4):558‐573. doi:10.1111/bpa.12698 30582229 PMC8028629

[25] Heverin M , Bogdanovic N , Lutjohann D , et al. Changes in the levels of cerebral and extracerebral sterols in the brain of patients with Alzheimer's disease. J Lipid Res. 2004;45(1):186‐193. doi:10.1194/jlr.M300320-JLR200 14523054

[26] Testa G , Staurenghi E , Zerbinati C , et al. Changes in brain oxysterols at different stages of Alzheimer's disease: their involvement in neuroinflammation. Redox Biol. 2016;10:24‐33. doi:10.1016/j.redox.2016.09.001 27687218 PMC5040635

[27] Papassotiropoulos A , Lutjohann D , Bagli M , et al. 24S‐hydroxycholesterol in cerebrospinal fluid is elevated in early stages of dementia. J Psychiatr Res. 2002;36(1):27‐32. 11755458 10.1016/s0022-3956(01)00050-4

[28] Wang H‐L , Wang Y‐Y , Liu X‐G , et al. Cholesterol, 24‐hydroxycholesterol, and 27‐hydroxycholesterol as surrogate biomarkers in cerebrospinal fluid in mild cognitive impairment and Alzheimer's disease: a meta‐analysis. J Alzheimers Dis. 2016;51:45‐55. doi:10.3233/JAD-150734 26836015 PMC6110663

[29] Papassotiropoulos A , Lutjohann D , Bagli M , et al. Plasma 24S‐hydroxycholesterol: a peripheral indicator of neuronal degeneration and potential state marker for Alzheimer's disease. Neuroreport. 2000;11(9):1959‐1962. doi:10.1097/00001756-200006260-00030 10884051

[30] Alzheimer's Association . 2022 Alzheimer's disease facts and figures. Alzheimers Dement. 2022;18(4):700‐789. doi:10.1002/alz.12638 35289055

[31] Shumaker SA , Legault C , Rapp S , et al. Estrogen plus progestin and the incidence of dementia and mild cognitive impairment in postmenopausal women: the Women's Health Initiative Memory Study: a randomized controlled trial. JAMA. 2003;289(20):2651‐2662. doi:10.1001/jama.289.20.2651 12771112

[32] Shumaker SA , Legault C , Kuller L , et al. Conjugated equine estrogens and incidence of probable dementia and mild cognitive impairment in postmenopausal women. JAMA. 2004;291(24):2947‐2958. doi:10.1001/jama.291.24.2959 15213206

[33] Dunk MM , Li J , Liu S , et al. Associations of dietary cholesterol and fat, blood lipids, and risk for dementia in older women vary by *APOE* genotype. Alzheimers Dement. 2023;19(12):1‐13. doi:10.1002/alz.13358 PMC10784407 37438877

[34] The Women's Health Initiative Study Group . Design of the Women's Health Initiative clinical trial and observational study. Control Clin Trials. 1998;19(1):61‐109. doi:10.1016/s0197-2456(97)00078-0 9492970

[35] Anderson GL , Manson J , Wallace R , et al. Implementation of the Women's Health Initiative study design. Ann Epidemiol. 2003;13:S5‐S17. doi:10.1016/S1047-2797(03)00043-7 14575938

[36] Chang P‐Y , Feldman D , Stefanick ML , et al. 27‐hydroxycholesterol, an endogenous SERM, and risk of fracture in postmenopausal women: a nested case‐cohort study in the Women's Health Initiative. J Bone Miner Res. 2019;34(1):59‐66. doi:10.1002/jbmr.3576 30138538 PMC6478389

[37] McDonald JG , Smith DD , Stiles AR , Russell DW . A comprehensive method for extraction and quantitative analysis of sterols and secosteroids from human plasma. J Lipid Res. 2012;53:1399‐1409. doi:10.1194/jlr.D022285 22517925 PMC3371252

[38] Friedewald WT , Levy RI , Fredrickson DS . Estimation of the concentration of low‐density lipoprotein cholesterol in plasma, without use of the preparative ultracentrifuge. Clin Chem. 1972;18(6):499‐502. 4337382

[39] Howie B , Fuchsberge C , Stephens M , Marchini J , Abecasis GR . Fast and accurate genotype imputation in genome‐wide association studies through pre‐phasing. Nat Genet. 2012;44(8):955‐959. doi:10.1038/ng.2354 22820512 PMC3696580

[40] Rapp SR , Legault C , Espeland MA , et al. Validation of a cognitive assessment battery administered over the telephone. J Am Geriatr Soc. 2012;60(9):1616‐1623. doi:10.1111/j.1532-5415.2012.04111.x 22985137 PMC3448122

[41] Morris JC , Heyman A , Mohs RC , et al. The consortium to establish a registry for Alzheimer's disease (CERAD): part I. Clinical and neuropsychological assessment of Alzheimer's disease. Neurology. 1989;39(9):1159‐1165. doi:10.1212/wnl.39.9.1159 2771064

[42] Petersen RC , Doody R , Kurz A , et al. Current concepts in mild cognitive impairment. Arch Neurol. 2001;58:1985‐1992. doi:10.1001/archneur.58.12.1985 11735772

[43] Dias IHK , Milic I , Lip GYH , et al. Simvastatin reduces circulating oxysterol levels in men with hypercholesterolaemia. Redox Biol. 2018;16:139‐145. 29501047 10.1016/j.redox.2018.02.014 PMC5952874

[44] Babiker A , Dzeletovic S , Wiklund B , et al. Patients with atherosclerosis may have increased circulating levels of 27‐hydroxycholesterol and cholestenoic acid. Scan J Clin Lab Invest. 2005;65:365‐376. doi:10.1080/00365510510025746 16081359

[45] Hughes TM , Kuller LK , Lopez OL . Markers of cholesterol metabolism in the brain show stronger associations with cerebrovascular disease than Alzheimer's disease. J Alzheimers Dis. 2012;39(1):53‐61. doi:10.3233/JAD-2012-111460 PMC3348402 22377780

[46] Trautwein EA , McKay S . The role of specific components of a plant‐based diet in management of dyslipidemia and the impact on cardiovascular risk. Nutrients. 2020;12:2671. doi:10.3390/nu12092671 32883047 PMC7551487

[47] Zhang X , Lv C , An Y , et al. Increased levels of 27‐hydroxycholesterol induced by dietary cholesterol in brain contribute to learning and memory impairments in rats. Mol Nutr Food Res. 2018;62:1700531. doi:10.1002/mnfr.201700531 29193679

[48] Sandebring‐Matton A , Goikolea J , Bjorkhem I , et al. 27‐hydroxycholesterol, cognition, and brain imaging markers in the FINGER randomized controlled trial. Alz Res Ther. 2021;13:56. doi:10.1186/s13195-021-00790-y PMC7937194 33676572

[49] O'Brien JT , Thomas A . Vascular dementia. Lancet. 2015;386(10004):1698‐1706. doi:10.1016/S0140-6736(15)00463-8 26595643

