# Supplementary material for: Plasma oxysterols are associated with serum lipids and dementia risk in older women
Source: Alzheimers Dement. 2024 Apr 4;20(5):3696–704. doi: 10.1002/alz.13811 (PMC11095475; doi:10.1002/alz.13811)
Supplement: Supplementary file 1 — Supporting Information [file ALZ-20-3696-s001.docx]

**APPENDIX A**

**Measurement of plasma oxysterols**

**Power calculations**

**Relationships of oxysterols with *APOE* and lipids**

**Table A1. Power calculations for multiple linear regression.**

**Relationships of oxysterols with cognitive outcomes**

**Table A2. Power calculations for Cox regression.**

**Figure A1. Distribution of oxysterols in the study sample.**

**Table A3.** **Associations between oxysterols and *APOE* status.**

**Figure A2. Associations between oxysterols and *APOE* status.**

**Figure A3. Associations between 24-OHC and lipids.**

**Figure A4. Associations between 27-OHC and lipids.**

**Figure A5. Associations between 24-OHC/27-OHC ratio and lipids.**

**Figure A6. Cumulative hazard curves for dementia and cognitive impairment based on varying levels of 24-OHC/27-OHC ratio by *APOE* status.**

**Supplementary analyses**

**Table A4. Associations between oxysterols and *APOE* status stratified by hormone therapy group.**

**Table A5. Associations between oxysterols and lipids stratified by hormone therapy group.**

**Table A6. Associations between oxysterols and dementia risk stratified by hormone therapy group.**

**Measurement of plasma oxysterols**

Banked EDTA plasma samples were shipped to UT Southwestern Department of Molecular Genetics (Dallas, TX).^35-37^ A modified Bligh-Dyer extraction was performed to isolate lipids in samples mixed with 300 μl of 10N potassium hydroxide (Fisher Scientific; Fair Lawn, NJ), 3 ml of 1:1 dichloromethane:methanol (dichloromethane from Honeywell - Burdick and Jackson; Morristown, NJ; methanol from Fisher Scientific), 20 μl of deuterated standards (Avanti Polar Lipids; Alabaster, AL), and 50 μg/ml of butylated hydroxytoluene (Sigma-Aldrich; St. Louis, MO).^36,37^ Aminopropyl solid-phase extraction (Biotage; Charlotte, NC) was used to isolate oxysterols.^36,37^ 24-OHC and 27-OHC were analyzed using a tertiary Shimadzu LC-20XR HPLC system (Shimadzu Scientific Instruments; Columbia, MD), which was coupled to an AB Sciex API-5000 MS equipped with Turbo V ESI source (Foster City, CA).^36,37^ A Kinetex C18 HPLC column (150 x 2.1 mm, 2.6 μm particle size; Phenomenex; Torrance, CA) was used to resolve oxysterols with a binary solvent gradient.^37^ The mobile phases were (A) 70% acetonitrile with 5 mM ammonium acetate (dissolved in water first), (B) 1:1 (v/v) acetonitrile:isopropanol with 5 mM ammonium acetate (dissolved first in isopropanol for 10-12 hours), and (C) dichloromethane.^36,37^

**Power calculations**

***Relationships of oxysterols with* APOE *and lipids***

Power calculations for multiple linear regression of plasma oxysterols in relation to *APOE* status and serum lipids were performed in G*Power version 3.1.9.4. Calculations were conducted according to both limited and full adjustment models. Power levels of 0.80 and 0.90 were used in these calculations, along with effect sizes of 0.15 and 0.35. The resulting estimates for adequate power in these models are shown in Table A1. With our current sample sizes for these analyses of 264 and 281, respectively, the calculations suggest sufficient power.

**Table A1. Power calculations for multiple linear regression.**

| **Independent variable** | **Covariate adjustment** | **Power** | **Effect size** | **Sample Size** |
| --- | --- | --- | --- | --- |
| *APOE* | Limited adjustment | 0.80 | 0.15 | 109 |
|  |  |  | 0.35 | 52 |
|  |  | 0.90 | 0.15 | 136 |
|  |  |  | 0.35 | 63 |
|  | Full adjustment | 0.80 | 0.15 | 143 |
|  |  |  | 0.35 | 70 |
|  |  | 0.90 | 0.15 | 175 |
|  |  |  | 0.35 | 84 |
| Blood lipids | Limited adjustment | 0.80 | 0.15 | 103 |
|  |  |  | 0.35 | 49 |
|  |  | 0.90 | 0.15 | 130 |
|  |  |  | 0.35 | 60 |
|  | Full adjustment | 0.80 | 0.15 | 139 |
|  |  |  | 0.35 | 68 |
|  |  | 0.90 | 0.15 | 171 |
|  |  |  | 0.35 | 81 |

***Relationships of oxysterols with cognitive outcomes***

Power calculations for Cox proportional hazards regression examining relations of plasma oxysterols with risk of dementia and cognitive impairment were calculated in the *powerSurvEpi* package in R. These calculations included the variance of each oxysterol (24-OHC=387.34, 27-OHC=1920.62, 24-OHC/27-OHC ratio=0.013) as well as the proportion of dementia (0.11) and cognitive impairment (0.19) cases. Estimated hazard ratios used in these calculations were 1.03 per ng/mL increase in 24-OHC and 27-OHC and 1.50 per unit increase in 24-OHC/27-OHC ratio. The sample sizes required to achieve a power of 0.80 or 0.90 for each oxysterol are presented in Table A2. Our sample size for these analyses was 328, suggesting that we had sufficient power. However, a larger sample size than is available in WHIMS is required for adequate power in the analysis of dementia and cognitive impairment risk in relation to 24-OHC/27-OHC ratio using Cox regression. This appears to be due to the small variance in 24-OHC/27-OHC ratio. Our findings are nevertheless informative given the limited research examining oxysterol-dementia relationships.

**Table A2. Power calculations for Cox regression.**

| **Oxysterol**  **(variance)** | **Cognitive outcome**  **(% of cases)** | **Estimated HR per unit**  **increase in oxysterol** | **Power** | **Sample size** |
| --- | --- | --- | --- | --- |
| 24-OHC | Dementia (0.11) | 1.03 | 0.80 | 196 |
| (387.34) |  | 1.03 | 0.90 | 271 |
|  | CI (0.19) | 1.03 | 0.80 | 114 |
|  |  | 1.03 | 0.90 | 157 |
| 27-OHC | Dementia (0.11) | 1.03 | 0.80 | 40 |
| (1920.62) |  | 1.03 | 0.90 | 55 |
|  | CI (0.19) | 1.03 | 0.80 | 23 |
|  |  | 1.03 | 0.90 | 32 |
| 24-OHC/27-OHC | Dementia (0.11) | 1.50 | 0.80 | 30,939 |
| (0.013) |  | 1.50 | 0.90 | 42,856 |
|  | CI (0.19) | 1.50 | 0.80 | 17,913 |
|  |  | 1.50 | 0.90 | 24,812 |

***Abbreviations:*** 24-OHC, 24(S)-hydroxycholesterol; 27-OHC, 27-hydroxycholesterol; CI, cognitive impairment (mild cognitive impairment + dementia combined); HR, hazard ratio.


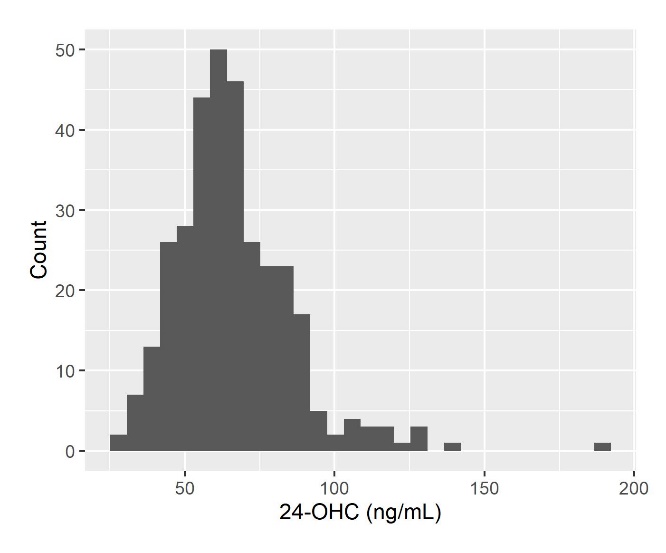

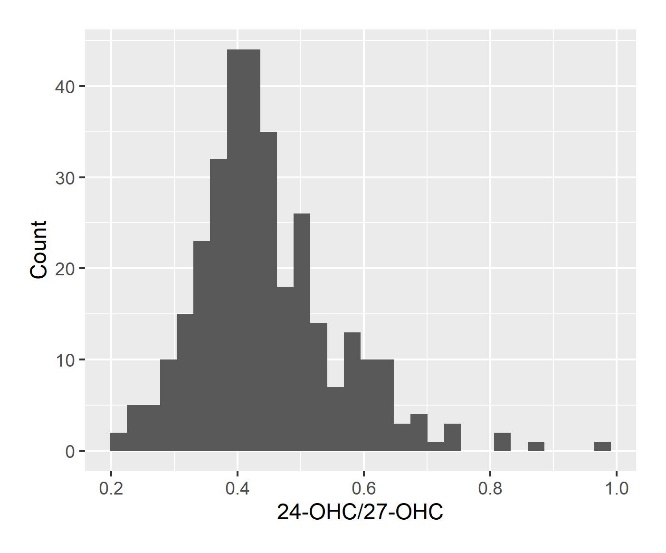

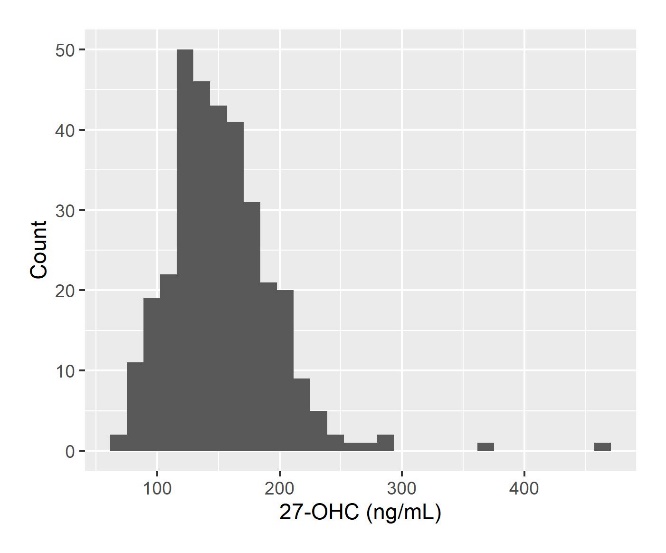


A B C

**Figure A1. Distribution of oxysterols in the study sample.** Histograms of (A) 24-OHC, (B) 27-OHC, and (C) 24-OHC/27-OHC ratio.

***Abbreviations*:** 24-OHC, 24(S)-hydroxycholesterol; 27-OHC, 27-hydroxycholesterol.

**Table A3.** **Associations between oxysterols and *APOE* status.**

|  | **Limited adjustment^+^ Full adjustment^‡^** | | | | |
| --- | --- | --- | --- | --- | --- |
| ***APOE*** | **LSM (SE)** | **β (95% CI)** | | **LSM (SE)** | **β (95% CI)** |
| **24-OHC** |  |  | |  |  |
| *APOE*2+ | 61.44 (3.31) | -4.07 (-10.55, 2.41) | | 60.01 (4.41) | -5.04 (-11.45, 1.36) |
| *APOE*3 | 65.51 (1.92) |  | | 65.06 (3.36) |  |
| *APOE*4+ | 65.70 (2.73) | 0.19 (-5.40, 5.78) | | 63.53 (3.91) | -1.52 (-7.10, 4.05) |
|  |  |  |  | |  |
| **27-OHC** |  |  | |  |  |
| *APOE*2+ | 140.55 (7.37) | -9.76 (-24.19, 4.67) | | 162.02 (9.94) | -8.48 (-22.92, 5.96) |
| *APOE*3 | 150.31 (4.28) |  | | 170.50 (7.58) |  |
| *APOE*4+ | 151.47 (6.08) | 1.16 (-11.29, 13.61) | | 166.32 (8.81) | -4.18 (-16.74, 8.38) |
|  |  |  |  | |  |
| **24-OHC/27-OHC** |  |  | |  |  |
| *APOE*2+ | 0.44 (0.02) | -0.01 (-0.04, 0.03) | | 0.38 (0.03) | -0.02 (-0.05, 0.02) |
| *APOE*3 | 0.45 (0.01) |  | | 0.39 (0.02) |  |
| *APOE*4+ | 0.45 (0.02) | 0.002 (-0.03, 0.03) | | 0.40 (0.02) | 0.01 (-0.03, 0.04) |

^+^Age, BMI, cholesterol-lowering medication, and HT trial assignment.

**^‡^**Age, BMI, cholesterol-lowering medication, HT trial assignment, education, hypertension, cardiovascular disease, diabetes, smoking, and weekly alcohol consumption.

***Abbreviations*:** 24-OHC, 24(S)-hydroxycholesterol; 27-OHC, 27-hydroxycholesterol; 95% CI, 95% confidence interval; BMI, body mass index; HT, hormone therapy; LSM, least squares means; SE, standard error.


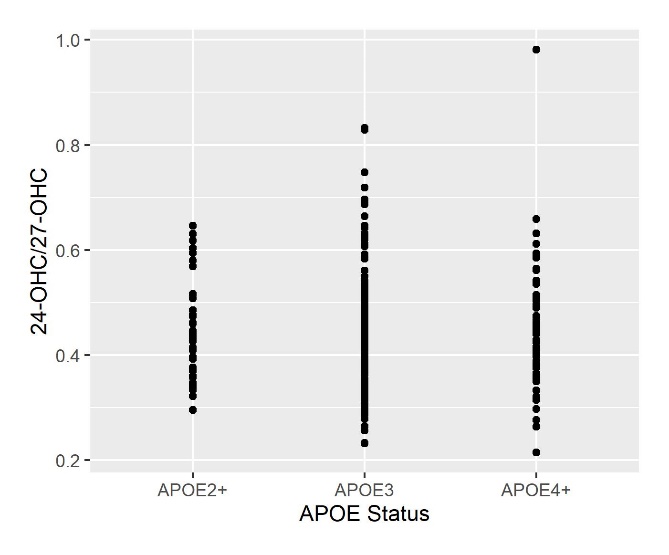

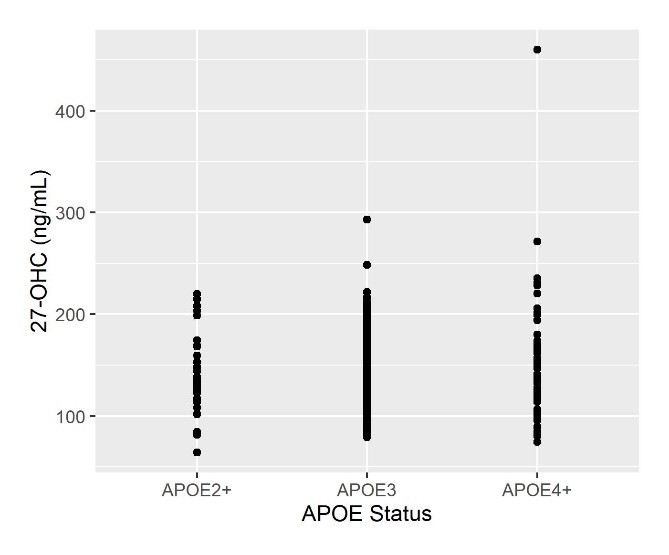

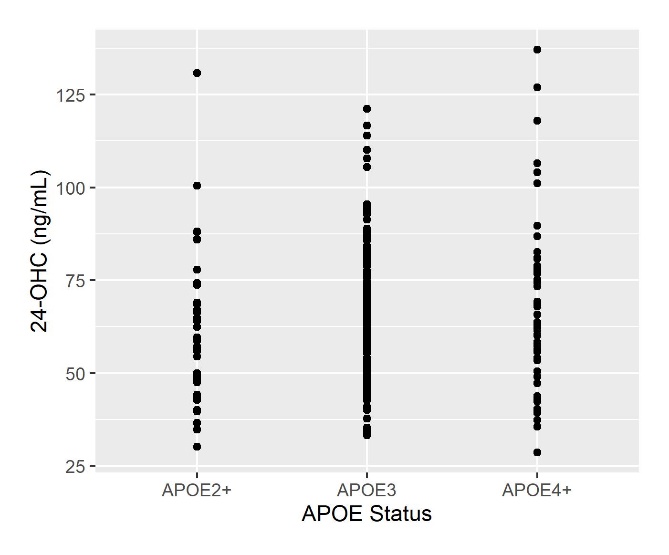


A B C

**Figure A2. Associations between oxysterols and *APOE* status.** Scatterplots of (A) 24-OHC, (B) 27-OHC, and (C) 24-OHC/27-OHC ratio by *APOE* status.

***Abbreviations*:** 24-OHC, 24(S)-hydroxycholesterol; 27-OHC, 27-hydroxycholesterol.

A B C D


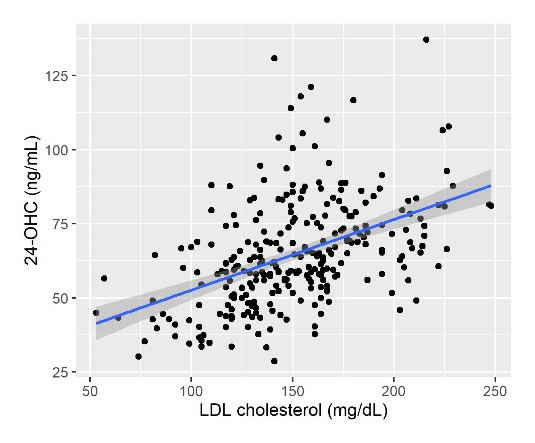

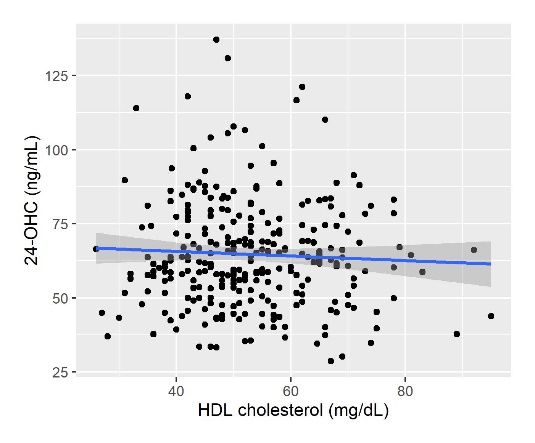

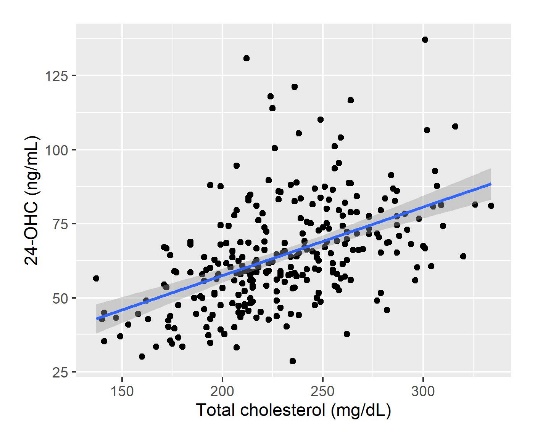

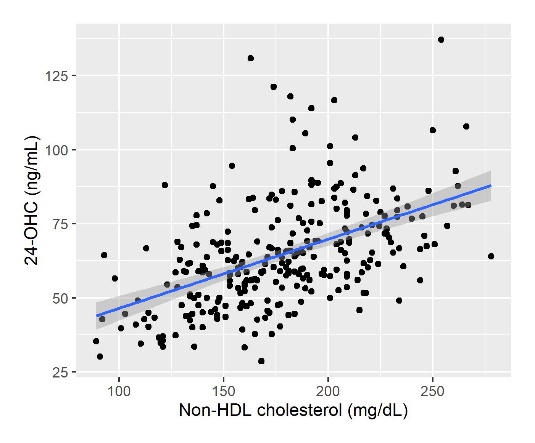

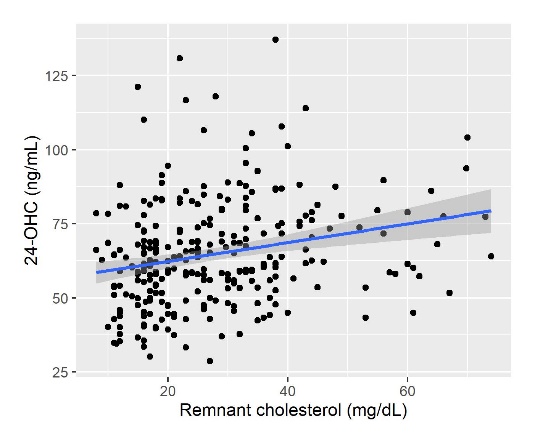

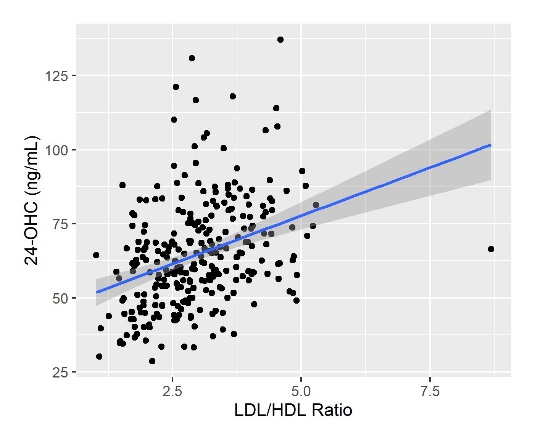

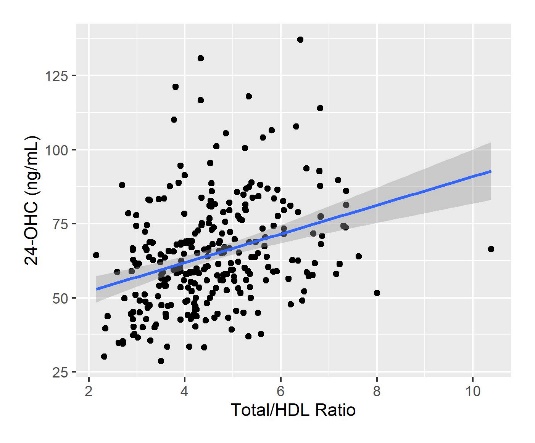

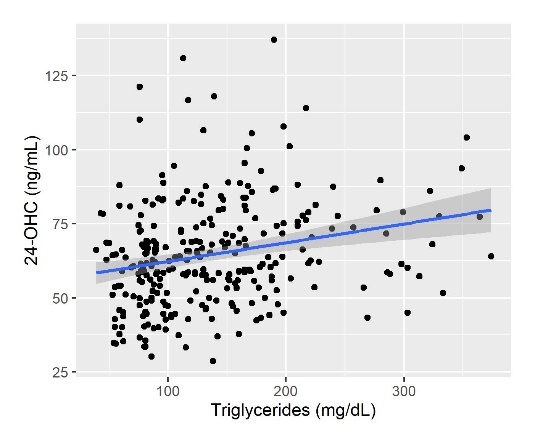


E F G H

**Figure A3. Associations between 24-OHC and lipids.** Scatterplots of 24-OHC by (A) LDL cholesterol, (B) HDL cholesterol, (C) total cholesterol, (D) non-HDL cholesterol, (E) remnant cholesterol, (F) LDL/HDL ratio, (G) total/HDL ratio, and (H) triglycerides.

***Abbreviations*:** 24-OHC, 24(S)-hydroxycholesterol; HDL, high-density lipoprotein; LDL, low-density lipoprotein.

A B C D


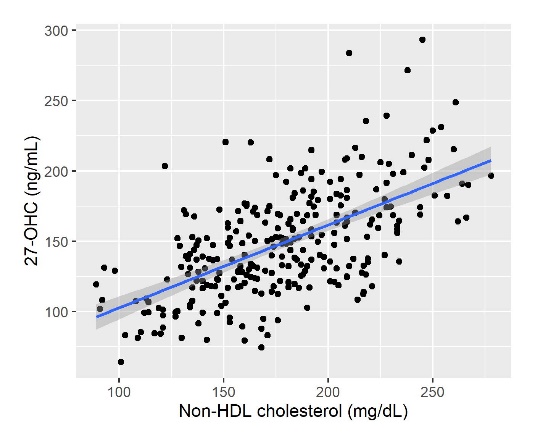

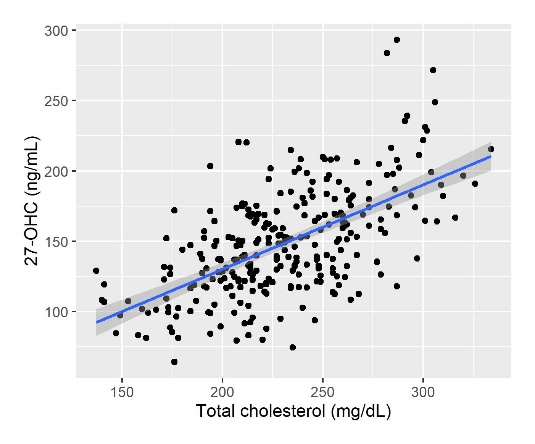

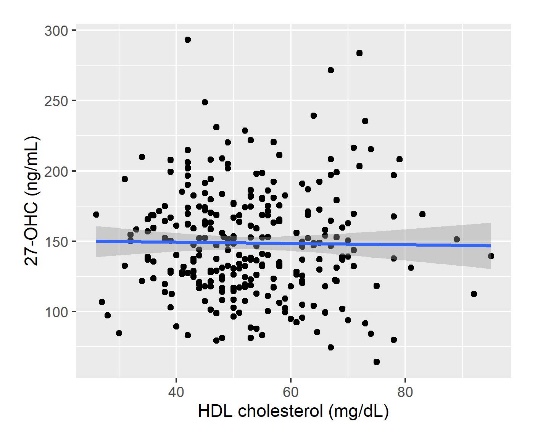

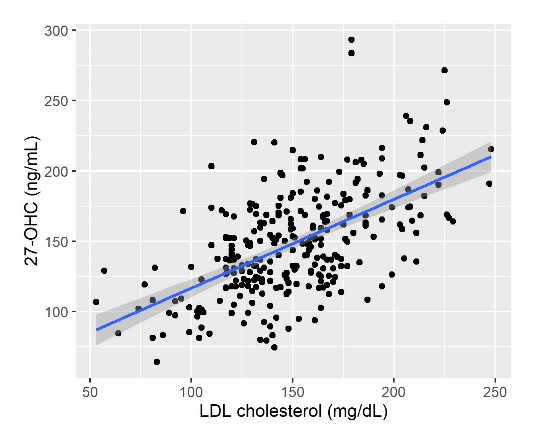

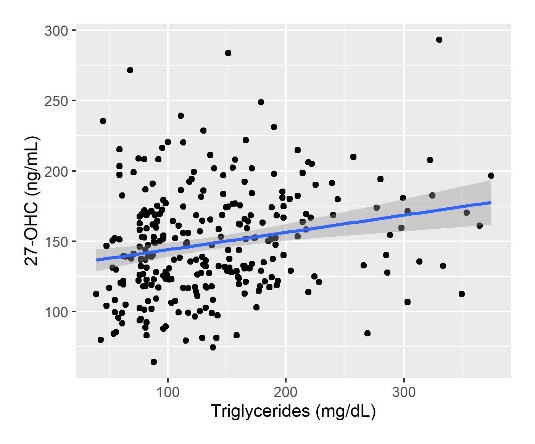

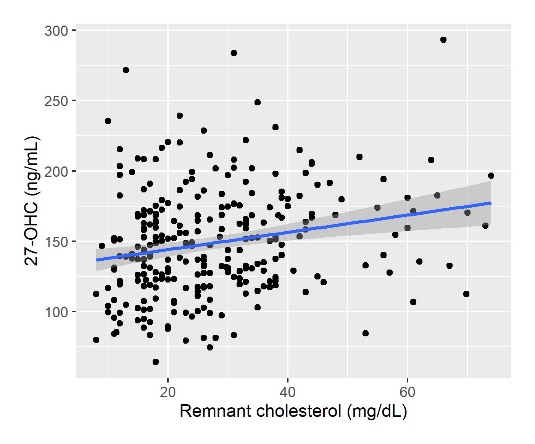

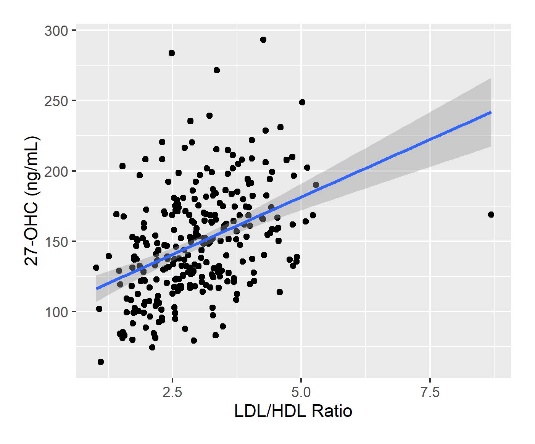

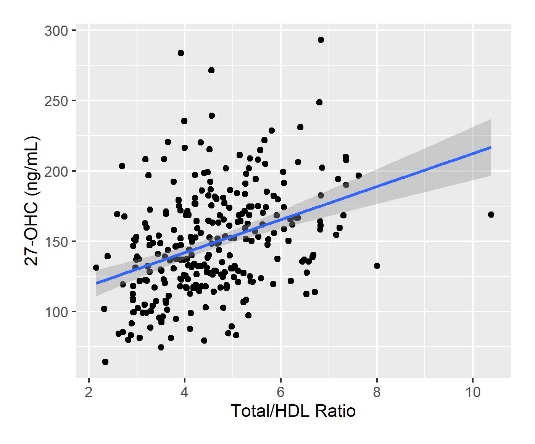


E F G H

**Figure A4. Associations between 27-OHC and lipids.** Scatterplots of 27-OHC by (A) LDL cholesterol, (B) HDL cholesterol, (C) total cholesterol, (D) non-HDL cholesterol, (E) remnant cholesterol, (F) LDL/HDL ratio, (G) total/HDL ratio, and (H) triglycerides.

***Abbreviations*:** 27-OHC, 27-hydroxycholesterol; HDL, high-density lipoprotein; LDL, low-density lipoprotein.

A B C D


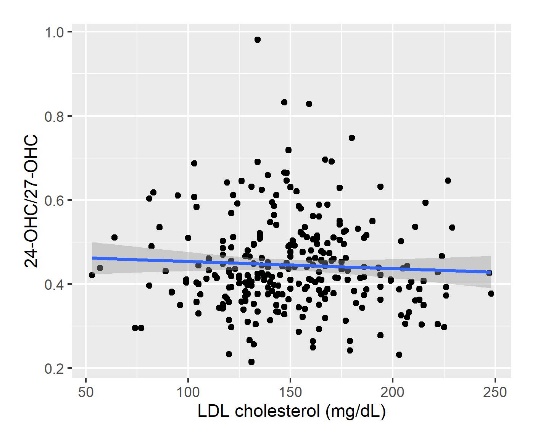

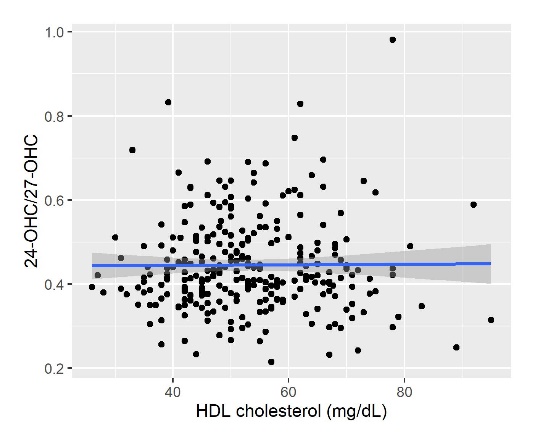

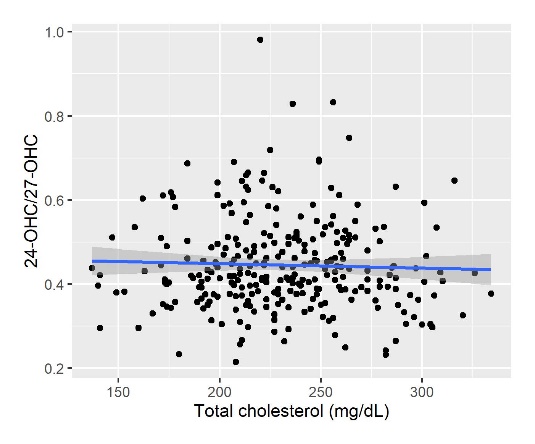

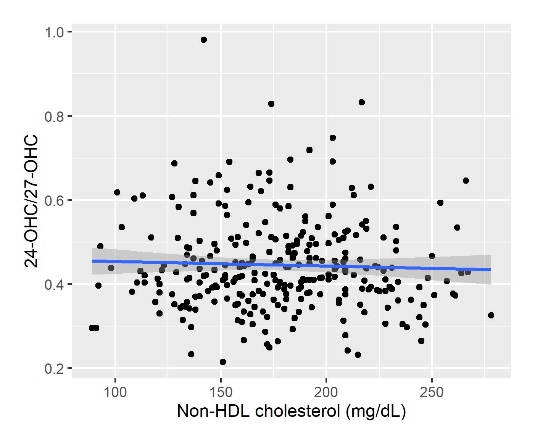

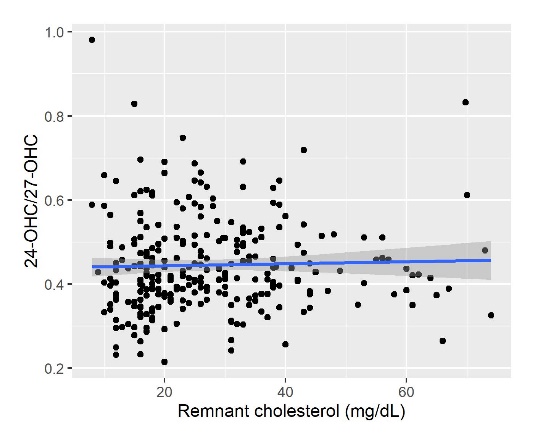

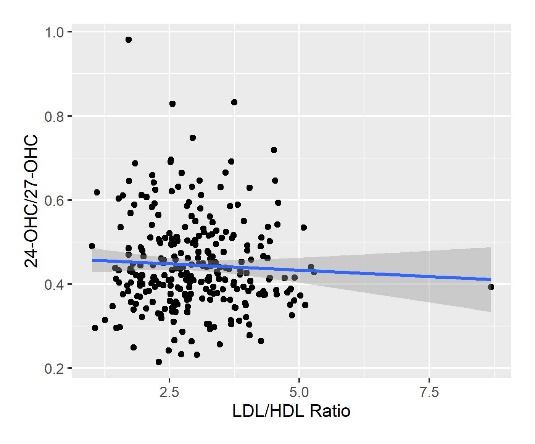

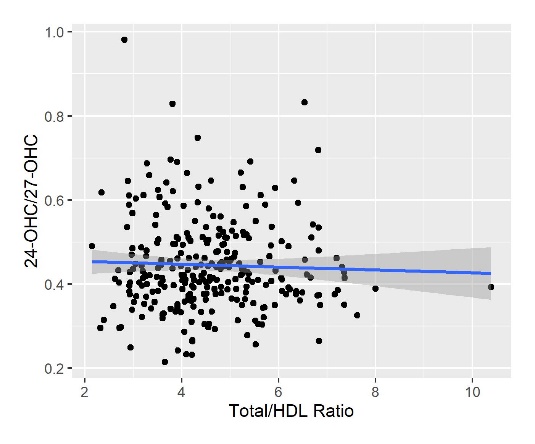

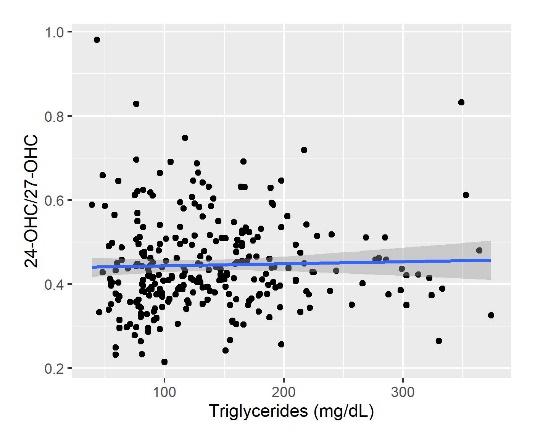


E F G H

**Figure A5. Associations between 24-OHC/27-OHC ratio and lipids.** Scatterplots of 24-OHC/27-OHC ratio by (A) LDL cholesterol, (B) HDL cholesterol, (C) total cholesterol, (D) non-HDL cholesterol, (E) remnant cholesterol, (F) LDL/HDL ratio, (G) total/HDL ratio, and (H) triglycerides.

***Abbreviations*:** 24-OHC, 24(S)-hydroxycholesterol; 27-OHC, 27-hydroxycholesterol; HDL, high-density lipoprotein; LDL, low-density lipoprotein.

***APOE*2+ carriers: *APOE*3 carriers: *APOE*4+ carriers:**

A B C


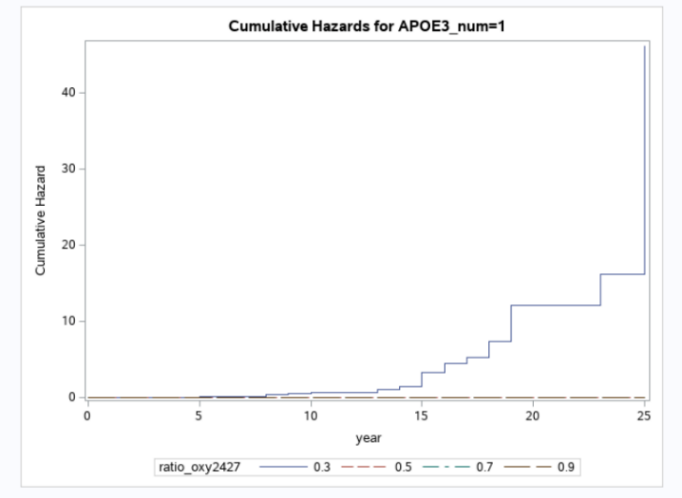

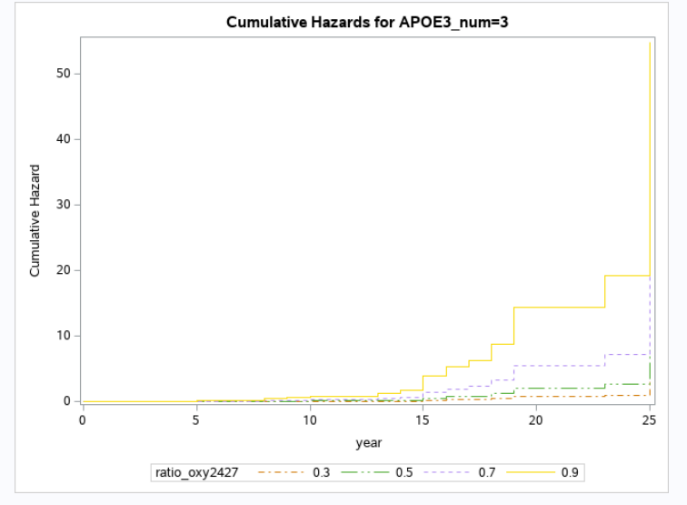

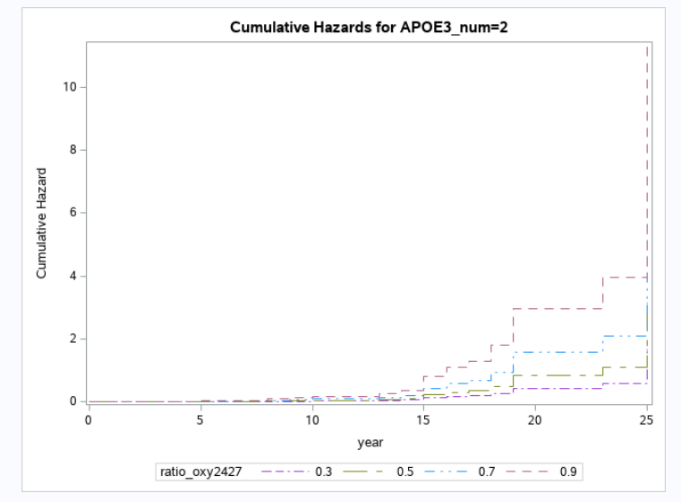


24-OHC/27-OHC Ratio

= 0.30

= 0.50

= 0.70

= 0.90

24-OHC/27-OHC Ratio

= 0.30

= 0.50

= 0.70

= 0.90

= 0.30

= 0.50, 0.70, 0.90

24-OHC/27-OHC Ratio

50

40

30

20

10

0

10

8

6

4

2

0

40

30

20

10

0

Cumulative Hazard of

Dementia

Cumulative Hazard of

Dementia

Cumulative Hazard of

Dementia

0 5 10 15 20 25

0 5 10 15 20 25

0 5 10 15 20 25

Years Since Randomization

Years Since Randomization

Years Since Randomization

30

20

10

0

D E F


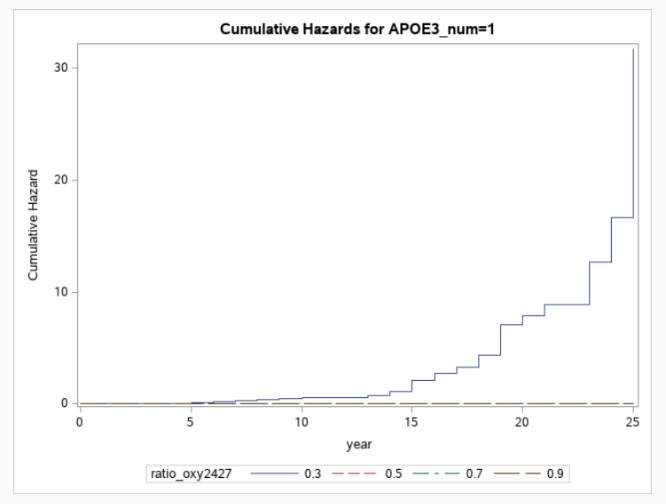

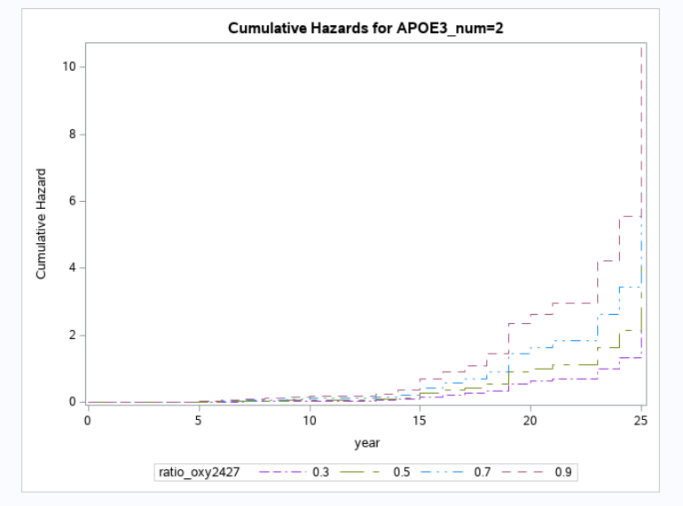

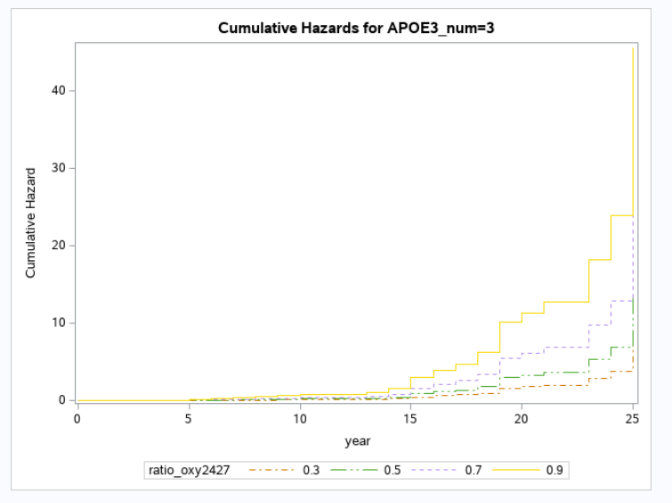


24-OHC/27-OHC Ratio

= 0.30

= 0.50

= 0.70

= 0.90

24-OHC/27-OHC Ratio

= 0.30

= 0.50

= 0.70

= 0.90

Cumulative Hazard of

Cognitive Impairment

40

30

20

10

0

Years Since Randomization

Years Since Randomization

Years Since Randomization

0 5 10 15 20 25

0 5 10 15 20 25

Cumulative Hazard of

Cognitive Impairment

10

8

6

4

2

0

= 0.30

= 0.50, 0.70, 0.90

24-OHC/27-OHC Ratio

0 5 10 15 20 25

Cumulative Hazard of

Cognitive Impairment

**Figure A6. Cumulative hazard curves for dementia and cognitive impairment based on varying levels of 24-OHC/27-OHC ratio by *APOE* status.** Cumulative hazard curves are shown at 24-OHC/27-OHC ratios of 0.30, 0.50, 0.70, and 0.90. Associations between 24-OHC/27-OHC ratio and cognitive outcomes are shown in graphs A and D for *APOE*2+ carriers, graphs B and E for *APOE*3 carriers, and graphs C and F for *APOE*4+ carriers. Graphs A-C depict cumulative hazard curves for dementia, and graphs D-F depict cumulative hazard curves for composite impairment (dementia+MCI).

**Supplementary analyses**

Associations between oxysterols and *APOE* remained non-significant in all hormone therapy groups (*p*’s≥0.08) (Table A4).

Associations between oxysterols and serum lipids stratified by hormone therapy are presented in Table A5. Among participants who received estrogen therapy (n=47), higher levels of LDL, total, non-HDL, remnant, LDL/HDL, and total/HDL cholesterol and triglycerides were associated with higher levels of 24-OHC and 27-OHC (*p*’s<0.0001). Higher HDL cholesterol was also associated with lower 24-OHC (*p*=0.01).

In the estrogen control group (n=75), higher levels of LDL, total, non-HDL, remnant, LDL/HDL, and total/HDL cholesterol and triglycerides were associated with higher 24-OHC (*p*’s<0.01). Higher LDL, total, non-HDL, LDL/HDL, and total/HDL cholesterol were also associated with higher 27-OHC (*p*’s<0.001). Additionally, higher levels of remnant cholesterol and triglycerides were associated with8 a higher 24-OHC/27-OHC ratio (*p*’s<0.05).

In the estrogen plus progestin intervention group (n=62), higher levels of LDL, total, non-HDL, LDL/HDL, and total/HDL cholesterol were associated with higher levels of both 24-OHC and 27-OHC (*p*’s<0.05).

Among the estrogen plus progestin control group (n=97), higher levels of LDL, total, non-HDL, LDL/HDL, and total/HDL cholesterol were associated with higher 24-OHC (*p*’s<0.05). Additionally, higher levels of LDL, total, non-HDL, remnant, LDL/HDL, and total/HDL cholesterol and triglycerides were associated with higher 27-OHC (*p*’s<0.05).

Following limited covariate adjustment, a higher 24-OHC/27-OHC ratio was associated with greater dementia (HR=2.11 [CI:1.18-3.76], *p*=0.01) and cognitive impairment (HR=1.95 [CI:1.29-2.95], *p*=0.002) risk in the estrogen plus progestin control group (n=110) (Table A6). Conversely, a higher 24-OHC/27-OHC ratio was associated with lower cognitive impairment risk following full adjustment in the estrogen control group (n=86; fully-adjusted HR=0.25 [CI:0.07-0.91], *p*=0.04). However, these results should be interpreted with caution due to the small sample sizes, and most fully-adjusted models failed to converge.

**Table A4. Associations between oxysterols and *APOE* stratified by hormone therapy group.**

|  |  | **Limited adjustment^+^** | | **Full adjustment^‡^** | |
| --- | --- | --- | --- | --- | --- |
| **HT assignment** | ***APOE*** | **LSM (SE)** | **β (95% CI)** | **LSM (SE)** | **β (95% CI)** |
| **Estrogen intervention, N=44** | **24-OHC** |  |  |  |  |
|  | *APOE*2+ | 55.38 (8.36) | -12.52 (-30.07, 5.04) | 50.32 (11.12) | -12.19 (-29.10, 4.72) |
|  | *APOE*3 | 67.90 (3.80) |  | 62.52 (7.76) |  |
|  | *APOE*4+ | 62.00 (5.82) | -5.90 (-18.77, 6.97) | 60.16 (9.12) | -2.35 (-15.65, 10.94) |
|  | **27-OHC** |  |  |  |  |
|  | *APOE*2+ | 133.22 (24.85) | -27.65 (-79.84, 24.55) | 145.63 (28.75) | -32.22 (-75.96, 11.51) |
|  | *APOE*3 | 160.87 (11.31) |  | 177.85 (20.06) |  |
|  | *APOE*4+ | 156.55 (17.30) | -4.32 (-42.58, 33.94) | 168.29 (23.60) | -9.56 (-43.95, 24.84) |
|  | **24-OHC/27-OHC** |  |  |  |  |
|  | *APOE*2+ | 0.41 (0.04) | -0.02 (-0.10, 0.06) | 0.37 (0.05) | -0.01 (-0.09, 0.06) |
|  | *APOE*3 | 0.43 (0.020 |  | 0.38 (0.03) |  |
|  | *APOE*4+ | 0.42 (0.03) | -0.01 (-0.07, 0.05) | 0.38 (0.04) | 0.01 (-0.05, 0.07) |
| **Estrogen control,**  **N=71** | **24-OHC** |  |  |  |  |
|  | *APOE*2+ | 63.43 (5.91) | -0.49 (-11.26, 10.29) | 62.27 (7.77) | -1.35 (-12.86, 10.17) |
|  | *APOE*3 | 63.92 (3.64) |  | 63.62 (5.80) |  |
|  | *APOE*4+ | 68.85 (4.50) | 4.94 (-3.67, 13.55) | 71.07 (5.72) | 7.45 (-1.81, 16.70) |
|  | **27-OHC** |  |  |  |  |
|  | *APOE*2+ | 148.18 (14.50) | -12.24 (-38.70, 14.23) | 158.15 (19.02) | -17.45 (-45.64, 10.74) |
|  | *APOE*3 | 160.42 (8.94) |  | 175.60 (14.19) |  |
|  | *APOE*4+ | 164.26 (11.05) | 3.84 (-17.30, 24.98) | 183.24 (14.01) | 7.64 (-15.01, 30.30) |
|  | **24-OHC/27-OHC** |  |  |  |  |
|  | *APOE*2+ | 0.46 (0.04) | 0.04 (-0.03, 0.11) | 0.43 (0.05) | 0.05 (-0.02, 0.12) |
|  | *APOE*3 | 0.42 (0.02) |  | 0.39 (0.03) |  |
|  | *APOE*4+ | 0.43 (0.03) | 0.02 (-0.04, 0.07) | 0.41 (0.03) | 0.02 (-0.04, 0.08) |
| **Estrogen plus progestin intervention, N=55** | **24-OHC** |  |  |  |  |
|  | *APOE*2+ | 59.58 (7.22) | -9.41 (-23.63, 4.81) | 49.40 (11.43) | -12.99 (-28.56, 2.58) |
|  | *APOE*3 | 68.99 (4.37) |  | 62.39 (9.14) |  |
|  | *APOE*4+ | 72.52 (7.55) | 3.53 (-11.40, 18.46) | 61.19 (11.70) | -1.21 (-16.61, 14.20) |
|  | **27-OHC** |  |  |  |  |
|  | *APOE*2+ | 141.89 (12.97) | -6.71 (-32.27, 18.85) | 145.41 (20.19) | -5.63 (-33.11, 21.86) |
|  | *APOE*3 | 148.60 (7.86) |  | 151.04 (16.15) |  |
|  | *APOE*4+ | 151.85 (13.57) | 3.25 (-23.58, 30.08) | 151.62 (20.67) | 0.59 (-26.62, 27.79) |
|  | **24-OHC/27-OHC** |  |  |  |  |
|  | *APOE*2+ | 0.41 (0.05) | -0.06 (-0.15, 0.03) | 0.31 (0.07) | -0.09 (-0.19, 0.01) |
|  | *APOE*3 | 0.47 (0.03) |  | 0.40 (0.06) |  |
|  | *APOE*4+ | 0.48 (0.05) | 0.01 (-0.09, 0.10) | 0.38 (0.08) | -0.02 (-0.12, 0.08) |
| **Estrogen plus progestin control,**  **N=94** | **24-OHC** |  |  |  |  |
|  | *APOE*2+ | 63.58 (5.73) | -0.18 (-11.34, 10.98) | 60.98 (7.64) | -1.24 (-11.72, 9.25) |
|  | *APOE*3 | 63.76 (3.46) |  | 62.22 (5.69) |  |
|  | *APOE*4+ | 61.14 (4.79) | -2.62 (-12.65, 7.41) | 57.32 (7.26) | -4.90 (-15.14, 5.34) |
|  | **27-OHC** |  |  |  |  |
|  | *APOE*2+ | 133.65 (10.84) | -4.15 (-25.29, 16.99) | 155.32 (15.03) | -0.73 (-21.36, 19.91) |
|  | *APOE*3 | 137.80 (6.55) |  | 156.05 (11.20) |  |
|  | *APOE*4+ | 137.33 (9.07) | -0.47 (-19.47, 18.53) | 146.76 (14.28) | -9.28 (-29.42, 10.86) |
|  | **24-OHC/27-OHC** |  |  |  |  |
|  | *APOE*2+ | 0.47 (0.03) | 0.003 (-0.06, 0.07) | 0.39 (0.05) | -0.01 (-0.08, 0.06) |
|  | *APOE*3 | 0.47 (0.02) |  | 0.41 (0.04) |  |
|  | *APOE*4+ | 0.47 (0.03) | -0.005 (-0.07, 0.06) | 0.42 (0.05) | 0.01 (-0.06, 0.08) |

^+^Age, BMI, and cholesterol-lowering medication.

**^‡^**Age, BMI, cholesterol-lowering medication, education, hypertension, cardiovascular disease, diabetes, smoking, and weekly alcohol consumption.

***Abbreviations*: 24-OHC**=24(S)-hydroxycholesterol; 27-OHC=27-hydroxycholesterol; 95% CI=95% confidence interval; BMI=body mass index; LSM=least squares means; SE=standard error.

**Table A5. Associations between oxysterols and lipids stratified by hormone therapy group.**

| **HT assignment** | **Lipid** | **Limited adjustment^+^** | | **Full adjustment^‡^** | |
| --- | --- | --- | --- | --- | --- |
|  |  | **β (95% CI)** | ***p*-value** | **β (95% CI)** | ***p*-value** |
| **Estrogen intervention,**  **N=47** | **24-OHC** |  |  |  |  |
|  | LDL cholesterol | 0.31 (0.17, 0.44) | <0.0001 | 0.30 (0.17, 0.42) | <0.0001 |
|  | HDL cholesterol | -0.36 (-0.74, 0.03) | 0.07 | -0.41 (-0.73, -0.08) | 0.01 |
|  | Total cholesterol | 0.28 (0.17, 0.39) | <0.0001 | 0.27 (0.16, 0.38) | <0.0001 |
|  | Non-HDL cholesterol | 0.31 (0.21, 0.41) | <0.0001 | 0.30 (0.21, 0.40) | <0.0001 |
|  | Remnant cholesterol | 0.71 (0.40, 1.03) | <0.0001 | 0.74 (0.43, 1.05) | <0.0001 |
|  | LDL/HDL ratio | 8.67 (3.99, 13.35) | 0.0003 | 7.85 (3.88, 11.82) | 0.0001 |
|  | Total/HDL ratio | 7.44 (3.81, 11.07) | <0.0001 | 6.72 (3.57, 9.86) | <0.0001 |
|  | Triglycerides | 0.14 (0.08, 0.21) | <0.0001 | 0.15 (0.09, 0.21) | <0.0001 |
|  | **27-OHC** |  |  |  |  |
|  | LDL cholesterol | 0.54 (0.26, 0.82) | 0.0001 | 0.65 (0.41, 0.89) | <0.0001 |
|  | HDL cholesterol | -0.31 (-1.10, 0.49) | 0.45 | -0.58 (-1.25, 0.08) | 0.09 |
|  | Total cholesterol | 0.48 (0.24, 0.72) | <0.0001 | 0.60 (0.40, 0.81) | <0.0001 |
|  | Non-HDL cholesterol | 0.49 (0.26, 0.72) | <0.0001 | 0.63 (0.45, 0.81) | <0.0001 |
|  | Remnant cholesterol | 0.82 (0.11, 1.53) | 0.02 | 1.35 (0.71, 1.98) | <0.0001 |
|  | LDL/HDL ratio | 15.91 (6.35, 25.47) | 0.001 | 18.36 (10.94, 25.77) | <0.0001 |
|  | Total/HDL ratio | 12.68 (5.10, 20.27) | 0.001 | 15.58 (9.74, 21.42) | <0.0001 |
|  | Triglycerides | 0.17 (0.02, 0.31) | 0.02 | 0.27 (0.14, 0.40) | <0.0001 |
|  | **24-OHC/27-OHC** |  |  |  |  |
|  | LDL cholesterol | 0.001 (-0.0004, 0.001) | 0.28 | 0.0002 (-0.001, 0.001) | 0.72 |
|  | HDL cholesterol | -0.002 (-0.004, 0.001) | 0.19 | -0.001 (-0.003, 0.001) | 0.35 |
|  | Total cholesterol | 0.001 (-0.0003, 0.001) | 0.22 | 0.0001 (-0.001, 0.001) | 0.85 |
|  | Non-HDL cholesterol | 0.001 (-0.0001, 0.001) | 0.10 | 0.0002 (-0.001, 0.001) | 0.60 |
|  | Remnant cholesterol | 0.002 (0.0002, 0.004) | 0.03 | 0.001 (-0.002, 0.003) | 0.52 |
|  | LDL/HDL ratio | 0.01 (-0.02, 0.04) | 0.42 | -0.0004 (-0.03, 0.03) | 0.98 |
|  | Total/HDL ratio | 0.01 (-0.01, 0.04) | 0.29 | -0.001 (-0.02, 0.02) | 0.95 |
|  | Triglycerides | 0.0004 (0.0000, 0.001) | 0.04 | 0.0001 (-0.003, 0.001) | 0.53 |
| **Estrogen control,**  **N=75** | **24-OHC** |  |  |  |  |
|  | LDL cholesterol | 0.20 (0.11, 0.30) | <0.0001 | 0.21 (0.12, 0.31) | <0.0001 |
|  | HDL cholesterol | -0.21 (-0.53, 0.11) | 0.19 | -0.12 (-0.50, 0.26) | 0.54 |
|  | Total cholesterol | 0.20 (0.12, 0.29) | <0.0001 | 0.21 (0.13, 0.29) | <0.0001 |
|  | Non-HDL cholesterol | 0.22 (0.14, 0.30) | <0.0001 | 0.23 (0.15, 0.31) | <0.0001 |
|  | Remnant cholesterol | 0.45 (0.20, 0.69) | 0.0004 | 0.49 (0.20, 0.77) | 0.001 |
|  | LDL/HDL ratio | 7.96 (4.31, 11.61) | <0.0001 | 9.32 (5.23, 13.42) | <0.0001 |
|  | Total/HDL ratio | 6.46 (3.67, 9.26) | <0.0001 | 8.32 (5.01, 11.63) | <0.0001 |
|  | Triglycerides | 0.09 (0.04, 0.14) | 0.0004 | 0.10 (0.04, 0.15) | 0.001 |
|  | **27-OHC** |  |  |  |  |
|  | LDL cholesterol | 0.07 (0.50, 0.96) | <0.0001 | 0.78 (0.56, 0.99) | <0.0001 |
|  | HDL cholesterol | -0.06 (-0.91, 0.80) | 0.89 | 0.75 (-0.28, 1.79) | 0.15 |
|  | Total cholesterol | 0.70 (0.50, 0.90) | <0.0001 | 0.73 (0.54, 0.92) | <0.0001 |
|  | Non-HDL cholesterol | 0.71 (0.51, 0.91) | <0.0001 | 0.72 (0.52, 0.92) | <0.0001 |
|  | Remnant cholesterol | 0.86 (0.17, 1.55) | 0.01 | 0.60 (-0.22, 1.43) | 0.15 |
|  | LDL/HDL ratio | 21.94 (12.26, 31.62) | <0.0001 | 23.56 (12.07, 35.05) | <0.0001 |
|  | Total/HDL ratio | 16.11 (8.51, 23.70) | <0.0001 | 17.64 (7.94, 27.34) | 0.0004 |
|  | Triglycerides | 0.17 (0.04, 0.31) | 0.01 | 0.12 (-0.04, 0.29) | 0.14 |
|  | **24-OHC/27-OHC** |  |  |  |  |
|  | LDL cholesterol | -0.001 (-0.001, 0.0001) | 0.11 | -0.001 (-0.001, 0.0000) | 0.07 |
|  | HDL cholesterol | -0.001 (-0.003, 0.001) | 0.40 | -0.002 (-0.004, 0.0001) | 0.07 |
|  | Total cholesterol | -0.0004 (-0.001, 0.0002) | 0.22 | -0.0004 (-0.001, 0.0002) | 0.18 |
|  | Non-HDL cholesterol | -0.0003 (-0.001, 0.0003) | 0.34 | -0.0003 (-0.001, 0.0003) | 0.36 |
|  | Remnant cholesterol | 0.001 (-0.001, 0.003) | 0.16 | 0.002 (0.0004, 0.004) | 0.01 |
|  | LDL/HDL ratio | -0.01 (-0.03, 0.02) | 0.67 | -0.001 (-0.03, 0.03) | 0.93 |
|  | Total/HDL ratio | 0.002 (-0.02, 0.02) | 0.88 | 0.01 (-0.01, 0.03) | 0.41 |
|  | Triglycerides | 0.0002 (-0.0001, 0.001) | 0.17 | 0.0004 (0.0001, 0.001) | 0.02 |
| **Estrogen plus progestin intervention,**  **N=62** | **24-OHC** |  |  |  |  |
|  | LDL cholesterol | 0.25 (0.12, 0.37) | <0.0001 | 0.25 (0.12, 0.38) | 0.0002 |
|  | HDL cholesterol | -0.02 (-0.41, 0.38) | 0.94 | -0.12 (-0.57, 0.32) | 0.59 |
|  | Total cholesterol | 0.23 (0.12, 0.34) | <0.0001 | 0.23 (0.11, 0.35) | 0.0002 |
|  | Non-HDL cholesterol | 0.23 (0.12, 0.34) | <0.0001 | 0.23 (0.11, 0.34) | 0.0001 |
|  | Remnant cholesterol | 0.23 (-0.10, 0.56) | 0.17 | 0.26 (-0.09, 0.61) | 0.15 |
|  | LDL/HDL ratio | 7.36 (2.38, 12.34) | 0.004 | 8.18 (2.95, 13.40) | 0.002 |
|  | Total/HDL ratio | 4.25 (0.65, 7.84) | 0.02 | 4.84 (1.03, 8.66) | 0.01 |
|  | Triglycerides | 0.05 (-0.02, 0.11) | 0.16 | 0.05 (-0.02, 0.12) | 0.14 |
|  | **27-OHC** |  |  |  |  |
|  | LDL cholesterol | 0.69 (0.51, 0.87) | <0.0001 | 0.69 (0.50, 0.88) | <0.0001 |
|  | HDL cholesterol | -0.14 (-0.85, 0.57) | 0.70 | -0.27 (-1.05, 0.51) | 0.50 |
|  | Total cholesterol | 0.60 (0.44, 0.76) | <0.0001 | 0.61 (0.43, 0.78) | <0.0001 |
|  | Non-HDL cholesterol | 0.61 (0.46, 0.77) | <0.0001 | 0.60 (0.43, 0.76) | <0.0001 |
|  | Remnant cholesterol | 0.48 (-0.10, 1.06) | 0.10 | 0.45 (-0.17, 1.06) | 0.16 |
|  | LDL/HDL ratio | 20.04 (12.02, 28.06) | <0.0001 | 20.87 (12.52, 29.22) | <0.0001 |
|  | Total/HDL ratio | 11.43 (5.38, 17.47) | 0.0002 | 12.03 (5.68, 18.38) | 0.0002 |
|  | Triglycerides | 0.10 (-0.02, 0.21) | 0.10 | 0.09 (-0.03, 0.21) | 0.15 |
|  | **24-OHC/27-OHC** |  |  |  |  |
|  | LDL cholesterol | -0.0003 (-0.001, 0.001) | 0.48 | -0.0003 (-0.001, 0.001) | 0.54 |
|  | HDL cholesterol | 0.001 (-0.002, 0.004) | 0.42 | 0.001 (-0.002, 0.004) | 0.66 |
|  | Total cholesterol | -0.0002 (-0.001, 0.001) | 0.61 | -0.0002 (-0.001, 0.001) | 0.68 |
|  | Non-HDL cholesterol | -0.0003 (-0.001, 0.001) | 0.45 | -0.0002 (-0.001, 0.001) | 0.60 |
|  | Remnant cholesterol | -0.0004 (-0.003, 0.002) | 0.73 | 0.0000 (-0.002, 0.002) | 0.98 |
|  | LDL/HDL ratio | -0.02 (-0.05, 0.02) | 0.33 | -0.01 (-0.05, 0.02) | 0.52 |
|  | Total/HDL ratio | -0.01 (-0.03, 0.01) | 0.37 | -0.01 (-0.03, 0.02) | 0.57 |
|  | Triglycerides | -0.0001 (-0.001, 0.0003) | 0.74 | 0.0000 (-0.0004, 0.001) | 0.97 |
| **Estrogen plus progestin control,**  **N=97** | **24-OHC** |  |  |  |  |
|  | LDL cholesterol | 0.26 (0.16, 0.35) | <0.0001 | 0.22 (0.12, 0.32) | <0.0001 |
|  | HDL cholesterol | -0.02 (-0.37, 0.34) | 0.93 | 0.12 (-0.23, 0.46) | 0.51 |
|  | Total cholesterol | 0.25 (0.16, 0.34) | <0.0001 | 0.21 (0.12, 0.30) | <0.0001 |
|  | Non-HDL cholesterol | 0.25 (0.16, 0.34) | <0.0001 | 0.20 (0.11, 0.29) | <0.0001 |
|  | Remnant cholesterol | 0.14 (-0.19, 0.47) | 0.41 | 0.10 (-0.22, 0.43) | 0.54 |
|  | LDL/HDL ratio | 6.27 (2.62, 9.92) | 0.001 | 4.25 (0.65, 7.86) | 0.03 |
|  | Total/HDL ratio | 4.75 (1.55, 7.95) | 0.004 | 3.21 (0.08, 6.35) | 0.04 |
|  | Triglycerides | 0.03 (-0.04, 0.09) | 0.41 | 0.02 (-0.04, 0.09) | 0.53 |
|  | **27-OHC** |  |  |  |  |
|  | LDL cholesterol | 0.63 (0.46, 0.79) | <0.0001 | 0.63 (0.46, 0.80) | <0.0001 |
|  | HDL cholesterol | -0.19 (-0.85, 0.48) | 0.59 | -0.08 (-0.77, 0.60) | 0.81 |
|  | Total cholesterol | 0.60 (0.44, 0.75) | <0.0001 | 0.60 (0.44, 0.75) | <0.0001 |
|  | Non-HDL cholesterol | 0.61 (0.46, 0.76) | <0.0001 | 0.60 (0.44, 0.75) | <0.0001 |
|  | Remnant cholesterol | 0.50 (-0.13, 1.13) | 0.12 | 0.67 (0.03, 1.31) | 0.04 |
|  | LDL/HDL ratio | 16.42 (9.86, 22.98) | <0.0001 | 14.95 (8.25, 21.65) | <0.0001 |
|  | Total/HDL ratio | 12.91 (7.11, 18.70) | <0.0001 | 12.21 (6.37, 18.05) | <0.0001 |
|  | Triglycerides | 0.10 (-0.03, 0.22) | 0.12 | 0.13 (0.01, 0.26) | 0.04 |
|  | **24-OHC/27-OHC** |  |  |  |  |
|  | LDL cholesterol | -0.0001 (-0.001, 0.001) | 0.78 | -0.0003 (-0.001, 0.0004) | 0.44 |
|  | HDL cholesterol | 0.001 (-0.001, 0.003) | 0.28 | 0.002 (-0.001, 0.004) | 0.12 |
|  | Total cholesterol | -0.0001 (-0.001, 0.001) | 0.83 | -0.0002 (-0.001, 0.0004) | 0.49 |
|  | Non-HDL cholesterol | -0.001 (-0.003, 0.001) | 0.38 | -0.0004 (-0.001, 0.0003) | 0.26 |
|  | Remnant cholesterol | -0.0002 (-0.001, 0.001) | 0.60 | -0.002 (-0.004, 0.001) | 0.17 |
|  | LDL/HDL ratio | -0.01 (-0.03, 0.01) | 0.38 | -0.02 (-0.04, 0.01) | 0.16 |
|  | Total/HDL ratio | -0.01 (-0.03, 0.01) | 0.31 | -0.02 (-0.04, 0.005) | 0.13 |
|  | Triglycerides | -0.0002 (-0.001, 0.0002) | 0.40 | -0.0003 (-0.001, 0.0001) | 0.18 |

^+^Age, BMI, and cholesterol-lowering medication.

**^‡^**Age, BMI, cholesterol-lowering medication, education, hypertension, cardiovascular disease, diabetes, smoking, and weekly alcohol consumption.

***Abbreviations:*** 24-OHC, 24(S)-hydroxycholesterol; 27-OHC, 27-hydroxycholesterol; 95% CI, 95% confidence interval; BMI, body mass index; HDL, high-density lipoprotein; HT, hormone therapy; LDL, low-density lipoprotein; LSM, least squares means; SE, standard error.

**Table A6. Associations between oxysterols and dementia risk stratified by hormone therapy group.**

| **HT assignment** | **Cognitive outcome** | **Limited adjustment^+^** | | **Full adjustment^‡^** | |
| --- | --- | --- | --- | --- | --- |
|  |  | **Hazard Ratio (95% CI)** | ***P*-value** | **Hazard Ratio (95% CI)** | ***P*-value** |
| **Estrogen intervention,**  **N=60,**  **PD=4,**  **CI=11** | **24-OHC** |  |  |  |  |
|  | PD | 1.03 (0.95, 1.12) | 0.46 | *Model did not converge* |  |
|  | CI | 1.02 (0.97, 1.07) | 0.53 | *Model did not converge* |  |
|  | **27-OHC** |  |  |  |  |
|  | PD | 1.01 (0.97, 1.06) | 0.49 | *Model did not converge* |  |
|  | CI | 1.01 (0.99, 1.04) | 0.40 | 1.07 (0.99, 1.15) | 0.12 |
|  | **24-OHC/27-OHC** |  |  |  |  |
|  | PD | 1.08 (0.30, 3.90) | 0.90 | *Model did not converge* |  |
|  | CI | 1.08 (0.53, 2.19) | 0.84 | 16.03 (0.35, 725.69) | 0.15 |
| **Estrogen control,**  **N=86,**  **PD=14,**  **CI=20** | **24-OHC** |  |  |  |  |
|  | PD | 1.00 (0.98, 1.03) | 0.80 | *Model did not converge* |  |
|  | CI | 1.00 (0.98, 1.03) | 0.76 | 0.98 (0.93, 1.03) | 0.34 |
|  | **27-OHC** |  |  |  |  |
|  | PD | 1.00 (0.99, 1.02) | 0.65 | *Model did not converge* |  |
|  | CI | 1.00 (0.99, 1.01) | 0.60 | 1.01 (0.99, 1.03) | 0.49 |
|  | **24-OHC/27-OHC** |  |  |  |  |
|  | PD | 0.89 (0.48, 1.67) | 0.73 | *Model did not converge* |  |
|  | CI | 0.90 (10.54, 1.50) | 0.69 | 0.25 (0.07, 0.91) | 0.04 |
| **Estrogen plus progestin intervention,**  **N=72,**  **PD=10,**  **CI=18** | **24-OHC** |  |  |  |  |
|  | PD | 1.01 (0.97, 1.04) | 0.71 | *Model did not converge* |  |
|  | CI | 1.01 (0.98, 1.03) | 0.68 | *Model did not converge* |  |
|  | **27-OHC** |  |  |  |  |
|  | PD | 1.00 (0.98, 1.02) | 0.87 | *Model did not converge* |  |
|  | CI | 1.00 (0.98, 1.01) | 0.71 | 0.95 (0.87, 1.05) | 0.34 |
|  | **24-OHC/27-OHC** |  |  |  |  |
|  | PD | 1.16 (0.66, 2.05) | 0.61 | *Model did not converge* |  |
|  | CI | 1.14 (0.75, 1.74) | 0.55 | *Model did not converge* |  |
| **Estrogen plus progestin control,**  **N=110,**  **PD=7,**  **CI=12** | **24-OHC** |  |  |  |  |
|  | PD | 1.01 (0.98, 1.05) | 0.45 | *Model did not converge* |  |
|  | CI | 1.02 (1.00, 1.05) | 0.12 | *Model did not converge* |  |
|  | **27-OHC** |  |  |  |  |
|  | PD | 0.99 (0.97, 1.01) | 0.35 | *Model did not converge* |  |
|  | CI | 1.00 (0.98, 1.01) | 0.46 | *Model did not converge* |  |
|  | **24-OHC/27-OHC** |  |  |  |  |
|  | PD | 2.11 (1.18, 3.76) | 0.01 | *Model did not converge* |  |
|  | CI | 1.95 (1.29, 2.95) | 0.002 | *Model did not converge* |  |

Hazard ratios of oxysterol levels were calculated per 1 ng/mL increase in 24-OHC and 27-OHC levels, and per 0.1 unit increase in 24-OHC/27-OHC ratio.

^+^Age, BMI, and cholesterol-lowering medication.

**^‡^**Age, BMI, cholesterol-lowering medication, education, cardiovascular disease, hypertension, stroke, diabetes, weekly alcohol consumption, smoking status, dietary cholesterol intake, total protein intake, total carbohydrate intake, total fat intake, total caloric intake, total blood cholesterol, and triglycerides.

***Abbreviations*:** 24-OHC, 24(S)-hydroxycholesterol; 27-OHC, 27-hydroxycholesterol; 95% CI, 95% confidence interval; BMI, body mass index; CI, cognitive impairment; HT, hormone therapy; PD, probable dementia.
